# Experiences of fathers of children with a life-limiting condition: a systematic review and qualitative synthesis

**DOI:** 10.1136/bmjspcare-2021-003019

**Published:** 2021-06-17

**Authors:** Victoria Fisher, Lorna Fraser, Johanna Taylor

**Affiliations:** Health Sciences, University of York, York, UK

**Keywords:** paediatrics

## Abstract

**Background:**

Children with a life-limiting condition often require extensive and complex care, much of which is provided by their parents at home. There is a growing body of research that aims to understand the experiences of these parents, but the majority of this research is from mothers’ perspectives, meaning that fathers’ experiences are not well understood.

**Objectives:**

To identify and synthesise findings from existing qualitative studies that have explored the experiences of fathers of children with a life-limiting condition.

**Methods:**

A systematic review of qualitative research was conducted using thematic synthesis. Searches were conducted in MEDLINE, CINAHL, EMBASE, PsycINFO and Social Science Citation Index.

**Results:**

Findings from 30 studies were included, representing the experiences of 576 fathers of children with a range of diagnoses including cancer, cystic fibrosis, genetic and neurological conditions. Themes detailed fathers’ experiences of uncertainty and shock around the time of their child’s diagnosis, their accounts of a ‘new normal’, difficulties in discussing their emotions, forming relationships with and seeking support from professionals and working fathers’ role conflicts. They discussed the life-changing nature of their child’s diagnosis, an event that affected all aspects of their lives from everyday activities, to their relationships, spirituality, values and ambitions.

**Conclusions:**

Fathers experience many difficulties in response to their child’s diagnosis and ongoing treatment. Findings highlight the need for healthcare professionals to recognise individual family dynamics and the evolving role of the father. Fathers’ responses are not widely understood, and research that directly addresses their own well-being is warranted.

**Key messages**

**What was already known?**

- Fathers are significantly under-represented in parental studies of children with a life-limiting condition.

**What are the new findings?**

- Fathers describe uncertainty related to their child’s condition and to their own role.
- They describe challenges in forming relationships with and seeking support from healthcare staff.

**What is their significance?**

- Healthcare professionals should be accommodating of fathers’ concerns and contributions to their child’s care as the role of the father evolves.
- Research that focuses on the mental health and well-being of these fathers is warranted.

## Background

Life-limiting conditions are those for which there is no reasonable hope of cure and from which a child or young person will eventually die, for example, Batten disease or Duchenne muscular dystrophy. Life-threatening conditions are those for which treatment may be feasible but can fail, for example, cancer or heart failure.^1^ From here on in, ‘life-limiting condition’ will refer to life-limiting and life-threatening conditions. Between 2001 and 2018 the prevalence of life-limiting conditions in children and young people in England increased from 26.7 per 10 000 to 63.2 per 10 000.^2^ Many of these children are dependent on medical technologies, such as ventilation,^3^ or gastrostomy feeds.^4^ Furthermore, they often require multiple medications either for the direct treatment of their condition or for pain and symptom management.^5^

In most cases, parents provide this complex and extensive care for their child, often with limited external support.^6^ Parental caregiving encompasses a range of responsibilities, including managing personal, emotional and medical care, advocacy in education and healthcare settings, researching the condition and treatment in order to inform medical decision-making, organising instrumental daily activities such as communication and transport, as well as ‘typical’ parental responsibilities.^7^ Palliative care forms an essential part of the care that some of these children receive, often from the point of diagnosis.^8^

Providing parents with support has long been recognised as being fundamental to paediatric palliative care^9^ and is well represented in guidelines for care professionals.^10^ The need for such is reflected in the growing body of research that focuses on the experiences and perspectives of parent caregivers. Existing studies focus largely on parental coping and quality of life,^11 12^ psychosocial outcomes,^13^ perceptions of and preferences for support^14 15^ and lived experiences,^16^ predominantly in oncology settings. Reviews have sought a more comprehensive understanding of current knowledge surrounding parental experiences, leading to new insights and recommendations for practice.^17^

However, a major limitation of this existing body of research is the under-representation of fathers in parental samples and the limited consideration of how fathers’ experiences or accounts may differ in studies that do include both mothers and fathers. For example, a recent meta-ethnography of the experiences of parents of children with a life-limiting condition^18^ included 17 studies; 6 of which focused solely on the experiences of mothers, 10 on those of both mothers and fathers and just 1 exclusively on the experiences of fathers. Furthermore, as recognised by the author, the mixed sample studies were heavily biased towards mothers.

From these accounts, we therefore know very little about how and why fathers experiences may differ, and any support or intervention for parents is likely to be founded on minimal input from fathers.^19^ Therefore, the overall aim of this systematic review and qualitative synthesis was to identify and synthesise qualitative findings related to the experiences of these fathers to understand issues that matter to them.

## Methods

### Study design

This review protocol was registered with PROSPERO CRD42020167076 (https://www.crd.york.ac.uk/prospero/display_record.php?RecordID=167076) and was reported in accordance with Enhancing Transparency in Reporting the synthesis of Qualitative research (ENTREQ) guidelines.^20^

### Eligibility criteria

#### Inclusion criteria

Studies were included if

- They used qualitative research methods to explore fathers’, step-fathers’, adoptive fathers’ or long-term foster fathers’ experiences of having a child of any age with a life-limiting condition diagnosed in childhood. This included bereaved fathers.
- ≥60% of the child population were diagnosed with a life-limiting condition as defined by the diagnoses listed in the search strategy.
- They used a mixed-methods approach and the qualitative data were reported separately and could be clearly extracted.
- They were published in English.

#### Exclusion criteria

Studies were excluded if

- They included the experiences of other participants that is, mothers or professionals.

### Search strategy

The search strategy was developed using terms for life-limiting conditions,^21^ father, children and a qualitative filter was applied (see Medline search strategy in online supplemental material). The SPIDER tool^22^ was used to define terms for each concept and advice was sought from an information specialist during initial development. Searches were run in electronic databases MEDLINE, CINAHL, EMBASE, PsycINFO and Social Science Citation Index from inception to 20 March 2020, using a combination of Medical Subject Headings (MeSH), keyword and free-text terms. Electronic searches were supplemented with citation searching, searching reference lists of included articles and a Google Scholar advanced search for grey literature. Searches were imported to Endnote,^23^ duplicates removed and uploaded to Covidence^24^ for screening. Title and abstract screening was undertaken by two reviewers and discussions were held after each 1000 studies had been screened. Any disagreements were resolved through discussion with a third reviewer. The full texts of potentially relevant studies were reviewed against the inclusion criteria by the same two reviewers and any disagreements resolved as above.

10.1136/bmjspcare-2021-003019.supp1 Supplementary data

### Data extraction

Key characteristics of each study, including authors, year of publication, year of data collection, country, setting, aims, methodology and methods and sample characteristics were extracted. All data labelled as ‘findings’, ‘results’ or pertaining to such, such as results reported in the discussion, were extracted to NVivo V.12.^25^ This included authors’ interpretations as well as quotes from participants.

### Quality appraisal

Each of the selected studies was subjected to quality appraisal using the Critical Appraisal Skills Programme,^26^ using a modified version of the tool^27^ to allow for greater clarity in assessing the philosophical position of studies and how this translated to their methods and methodologies. Studies were given a rating of ‘high’, ‘medium’ or ‘low’ quality though no studies were excluded on the basis of quality as this has not been shown to improve the quality of the review and may lead to unwarranted exclusions.^28^

### Synthesis

Thematic synthesis^29^ was selected due to its accessibility, its ability to synthesise heterogeneous studies from a range of epistemological positions and its suitability for exploring under-researched areas.^30^ Text was coded line-by-line to create a bank of codes and concepts that were translated across studies. More than one code could be assigned to a line and new codes were added when necessary. Descriptive themes were developed by grouping codes together based on similarities and analytical themes were developed to ‘generate new interpretive constructs, explanations or hypotheses’.^29^ Articles were each analysed to the same extent and in an order determined by diagnostic category to explore potential differences between these throughout the analysis. For example, studies on fathers of children with cancer, who are often studied as a distinct population and viewed as qualitatively different to children with other life-limiting conditions, were coded last, in order to assess whether these studies added any significantly different concepts to the coding structure. An assessment was also made as to the extent to which lower-quality studies contributed to the coding structure and theme development. Coding was carried out by one reviewer (VF) with regular input from the review team during the development of descriptive and analytical themes.

## Results

### Identification and selection of studies

**Figure 1:**
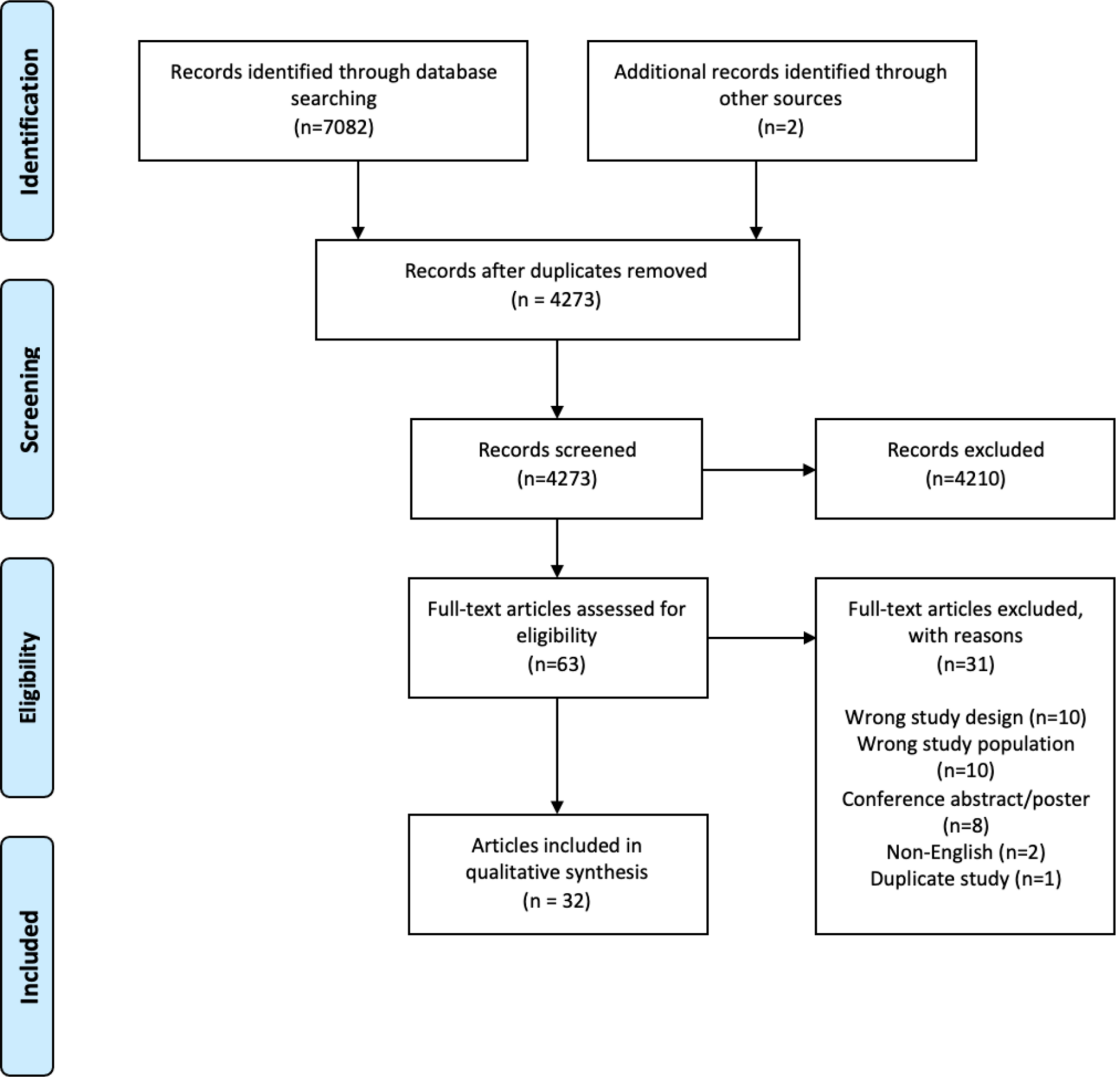
Preferred Reporting Items for Systematic Reviews and Meta-Analyses flowchart showing the inclusion of 32 studies from the 7082 identified.

The electronic database searches identified 4273 unique results. A total of 4210 studies were excluded during title and abstract screening leaving 61 papers, that were assessed against the eligibility criteria during the full-text screening. Thirty-one papers were excluded during full-text screening leading to the inclusion of 32 papers from 30 studies (figure 1).

### Characteristics of included studies

Studies were published between 1997 and 2019 with data collection taking place between 1978 and 2019. The majority of the included papers were published in the USA (17), followed by the UK (4), Canada (3) and Australia (2). There was one paper each from New Zealand, Ireland, Hong Kong, Sweden and Brazil. There were 27 papers from peer-reviewed journals, 5 were PhD theses (online supplemental table 1).

10.1136/bmjspcare-2021-003019.supp2 Supplementary data

All but one study used semistructured interviews for data collection, and the others used focus groups. Where stated, methodologies included phenomenology, narrative approach, grounded theory, life-story and mixed methods. Most studies employed a form of thematic or content analysis.

### Characteristics of participants and their children

The included studies represented the experiences of 576 fathers including biological fathers, stepfathers and adoptive fathers. This included bereaved fathers. Sample sizes for studies generally ranged from 6 to 24, though three studies included larger sample sizes of 60, 63 and 167. From the demographics that were reported, the age range of the fathers was 23–65 years. They came from a range of educational backgrounds and occupations. The majority were married/cohabiting with the mother of their child/children and were in employment (online supplemental table 1).

Fifteen articles (from 13 studies) focused on fathers of children with cancer,^31–45^ five on fathers of children with a congenital heart defect,^46–50^ three on fathers of children with cystic fibrosis,^51–53^ two on fathers of children with neurological conditions^54 55^ and one focused on fathers of children with a genetic condition.^56^ Six studies were not diagnosis specific and included fathers of children with a range of life-limiting and life-threatening conditions.^57–62^ The children were aged between infancy and 27 years but were all diagnosed in childhood (online supplemental table 1).

### Quality appraisal

The majority of the studies were of medium to high quality (online supplemental information). The majority of studies had clear aims and objectives for which qualitative designs were suitable. Sampling strategies and data collection methods were generally well defined and appropriate for the research area. Reflexivity was poorly discussed in many studies and the relationship between researcher and participant was not always clear.

### Results of thematic synthesis

Line-by-line coding led to the development of 245 codes (examples of which can be seen in table 1). Similarities between codes were identified and which were then grouped into 10 descriptive themes. The descriptive themes were synthesised further to develop four analytical themes (table 1), which are described below and illustrated with quotes from the included studies.

**Table 1:** Relationship between codes, descriptive themes and analytical themes

Analytical themes	Descriptive themes	Codes
Uncertainty: introduction and adaptation	Experience of diagnosis Coping with diagnosis Thinking about the future	Feeling helpless during diagnostic period Waiting for diagnosis Diagnosis did not seem real Diagnosis brings uncertainty Planning ahead Identifying cause Trying to take control Waiting for appointments Seeking prognosis Knowledge helps to feel safe
A new normal	Relationships with family and friends Values and perspectives Day-to-day life	A lot of travel Constant presence of illness Condition continually demanding Constant hypervigilance Disruptions to everyday life Spontaneity is difficult Others do not understand Relationship with partner became stronger Conflict with partner Lack of support from friends and family
Professionals reinforcing fathers’ role perceptions	Emotional experiences Sharing of emotions and support Relationship with professionals	Lack of sensitivity from healthcare professionals Lasting emotional impact of hospital experience Lack of information Need reassurance from professionals Treated differently to mothers Lack of recognition of emotional strain Support more readily available for mothers Perceived as being the strong parent Need to be strong for others Loneliness for fathers
Working fathers: role conflict	Workplace experiences	Career adaption due to diagnosis Able to work remotely Lack of understanding at work Colleagues understanding Unable to concentrate Loss of employment Work restricts appointment attendance Pressure to succeed at work Opportunities for support restricted by work Would like more flexibility at work Pressures at home affected work

### Uncertainty: introduction and adaptation

#### New uncertainty

**Figure 2:**
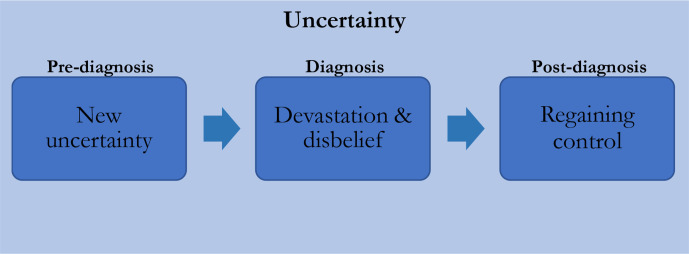
Subthemes of analytical theme 1.

Uncertainty dominated fathers’ accounts of their child’s condition. Three subthemes (figure 2), organised temporally, form this theme; the introduction of a new uncertainty prediagnosis, the transition to a new form of uncertainty at diagnosis and finally fathers’ adjustment to this uncertainty post diagnosis.

The period before diagnosis varied for fathers, though many endured difficult ‘battles’, multiple hospital visits, demanding conversations and long waits in order to get a confirmed diagnosis.^31 33 36 55 57 60^ Fathers gave several reasons for this delay, including a lack of existing knowledge surrounding their child’s condition. This delay caused frustration, deepened fathers’ uncertainties and often left them feeling helpless.

Delay in diagnosis was due to several causes: inadequate medical resources; current state of knowledge of childhood cancers; and, in one case, turf issues between medical personnel.^36^ (quote from study authors, cancer, USA)

The ‘delay’ and long time that passed between the onset of symptoms and diagnosis, combined with various exams the child has to take, generate anxiety, anguish, and uncertainty.^55^ (quote from study authors, Duchenne Muscular Dystrophy, Brazil)

#### Diagnosis: devastation and disbelief

Diagnosis was shocking for fathers^31 33 36 40 42 54–57 60^ with many unable to comprehend the reality of what had happened.^34 40 43 52 54 57 58 60^ Despite some diagnoses confirming fathers’ expectations, this sense of shock was prominent in their accounts, perhaps demonstrating a sense of hope leading up to diagnosis. Fathers expressed a range of negative emotions such as sadness,^38 48^ anger,^38 56 57^ devastation,^34 36 52 57 60^ helplessness^33 34 43 44 48 55 58^ and described feeling overwhelmed.^53 60^ Some described feelings of grief and loss over their child’s future,^36 42 54 55 60 62^ and loss of their expected experience of fatherhood.^60^ Fathers described diagnosis as both a surreal but traumatic event; like being ‘thrown into a hurricane’,^62^ like a ‘bomb’^55^ and like being ‘smacked in the guts with a sledgehammer’.^34^

You feel that you’ve been taken out of your life and put into somebody else’s movie…the wrong movie. Like if you were watching a film in the theatre and when they change the reels, they put on a reel from a different movie…it’s that disorienting. It’s a very alienating experience.^58^ (life-limiting conditions, USA)

A feeling of devastation, yes, you know that somehow the world changed from what it was an hour and a half before.^60^ (quote from participant, life-limiting condition, UK)

#### Regaining control

The uncertainty that fathers experienced in the period leading up to their child’s diagnosis, evolved at diagnosis. Here, uncertainties related to their child’s condition and treatment, including potential causes of illness, and their child’s future. Fathers struggled to make sense of the diagnosis and information seeking played a part in their behavioural response to such.^36 41^ For some there was an initial, transient form of information seeking, for some to find an explanation for or seeking to attribute blame for their child’s condition.^34 36 40 45 56 58^

We had a dog, did the kid play with the dog too much?!’ Some wondered if they themselves had done something wrong: ‘I did construction on the house. Did I bring some contaminated material into the house?^58^ (quote from study participant, life-limiting conditions, USA)

I blamed myself because I should have told him not to eat cup noodles for lunch every day. The preservatives might have caused his disease.^40^ (quote from study participant, cancer, Hong Kong)

Other information seeking related to prognosis, trajectory and living with the condition, and this helped some fathers to assert some control in areas they felt could be managed. Fathers straightforwardly described focusing on ‘what needed to be done’,^31^ taking ‘one day at a time’,^38^ the ‘here and now’,^52^ ‘tackling things head on’,^59^ ‘getting on with it’^43^ and a determination that the illness could be ‘beaten’.^62^ Many fathers also described their faith or religion as being important throughout this period and beyond,^31 35 38 53 54 58^ the importance of which sometimes heightened as a result of their child’s illness. Fathers sought to regain control through understanding and began to consider that uncertainty would be a part of their lives, and despite the unsettling nature of this uncertainty, accepting and adapting.

…in the last few years I've found I became more positive you know, trying to…not live with kind of a cure, just trying to live with it…the bottom line is…none of us know what is going to happen to us tomorrow anyhow.^51^ (quote from study participant, cystic fibrosis, Ireland)

One way in which the fathers dealt with the unpredictability of the disease and their lack of control over it was to set their sights on more immediate and achievable goals, such as becoming the advocate of their child. 36 (quote from study authors, cancer, USA)

### A new normal

Fathers’ lives were often consumed with fighting or ‘battling’ their child’s illness,^35 36 43 47 55 58^ so much so that relationships, daily activities, priorities, values and spirituality were all affected to create a ‘new normal’.

Fathers discussed the transformative nature of illness on their relationships with family and friends. Many worried about their partners, who were most often the primary caregiver. Fathers talked about their concern for their family’s well-being and a desire to support and protect them,^35 38 41 43 50^ by being ‘the rock of the family’.^39^ This concern for and the prioritisations of others, could leave fathers feeling lonely and isolated. Some relationships became practical, with many couples only seeing one another while taking over from one another in their child’s care at hospital.^38 42 44 57^ Some fathers described their relationships as growing stronger, with many fathers expressing gratitude for and pride in their partner.^33 41–43 54^

…fathers experience themselves as being in a battle for their child’s and their family’s health and well-being, a perception that arise when looking for possibilities to be together, get information about the child’s disease, and be involved in the child’s care. During such times, fathers struggle to cope with their own experiences and feelings.^47^ (quote from study authors, congenital heart defect, Ireland)

Relationships with extended family members and friends were also affected and resulted in shifts in fathers’ social circles. Much of this was related to fathers seeking those with a shared understanding, that was not present in their existing relationships, for example through support groups.^32 35 38 42–44^

Some parents experience insensitivity and avoidance from friends when dealing with their child’s illness (Patterson et al., 2004), increasing the importance of finding other parents who share their circumstances.^31^ (quote from study authors, cancer, USA)

Some fathers also described positive transformations in regard to their outlook on life. They discovered new meanings in life and reassessed their priorities and values. Some discovered the meaning of community after receiving financial, emotional and practical support from their local communities.

It’s just been a heck of a ride. But I’m very grateful for the fact that she is still alive today, and she’s a beautiful young lady. 54 (quote from study participant, cerebral palsy, USA)

### Professionals’ reinforcing fathers’ role perceptions

**Figure 3:**
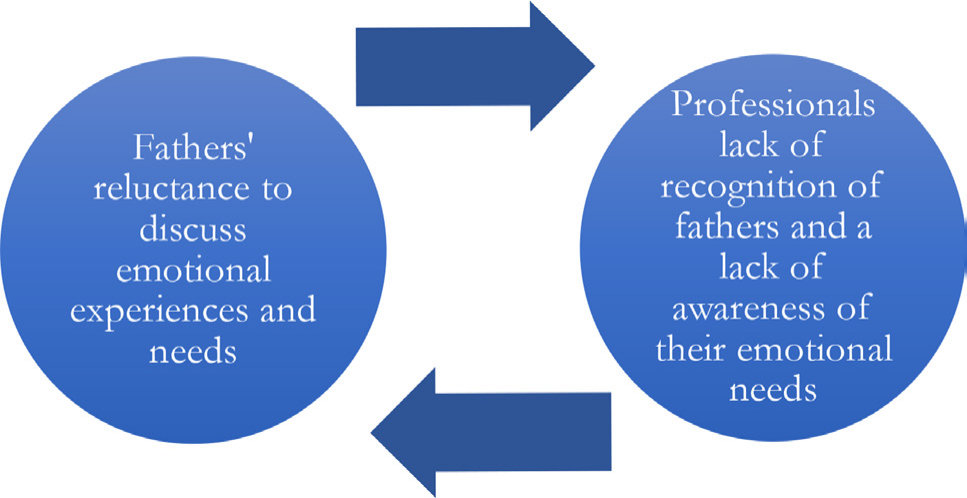
Reciprocal relationship between subthemes of theme 3.

This theme incorporated two concepts: how fathers perceived their role and emotional needs, and how this was often reinforced through their interactions with healthcare professionals. Figure 3 demonstrates the relationship between these subordinate concepts.

#### Keeping emotions hidden from others

Fathers experienced a range of emotions and challenges in their everyday lives at, and beyond, diagnosis. Fathers discussed the overwhelming and turbulent nature of their emotional worlds.^31 32 57 59^ They described mental strain,^31^ exhaustion,^33 42 44 47 49 56^ loneliness,^32 36 41 42 44 47 53 59 60^ vulnerability^47 58^ and sadness.^32 33 41 47 59^ Fathers described an underlying anxiety related to a fear of bad news,^32 48 51 59^ the trajectory of the illness^49 51 56^ and relapse.^42^

Liam described how he is affected emotionally by the illness spontaneously and acknowledges how his emotional world affects his thoughts about the future: I get really upset about it sometimes often at the most random time yeh you’d be having a completely normal time and then all of a sudden it hits you oh my god.^57^ (quote from study authors and participant, life-limiting conditions, UK)

Some fathers also described the constant and underlying fear they had of exposing their child to unnecessary risk or missing important symptoms, resulting in a state of hypervigilance and further contributing to their exhaustion.^31 33 36 37 42 43 49 51 57^

There was a sense of exhaustion from constant hyper-vigilance while taking responsibility for their child’s safety and being the omnipotent protector proved a tough task, from which it was difficult to escape.^49^ (quote from study authors, congenital heart defects, UK)

Fathers discussed keeping these emotional struggles hidden from their families out of a concern that they would cause further suffering to those around them.^32 35 42–44 48–52 55 57 59 60 62^ In focusing on the needs of others fathers were able to deflect from their own experiences and minimise their own suffering^36 37 43 44 49 51 55 57 60^; to discuss their distress would be too difficult and they did not have the tools to do so.^32 44 51 55 57^

It may have also served as protection from potentially overwhelming emotions which could jeopardise the role of container; perhaps a focus on practicalities provided an escape from their own emotional responses so they could avoid upsetting others.^49^ (quote from study authors, congenital heart defect, UK)

Conversely the need for and the value of support was recognised, sometimes in hindsight:

You can bury down as long as you want. You can push it (emotion) away and try not think about it or just ignore it. But, eventually, it is going to come back to the surface. Deal with it.^39^ (quote from study participant, cancer, USA)

#### Fathers: the forgotten parent in healthcare settings

In prioritising the needs of their families, fathers positioned themselves as supportive figures. On reflection, some fathers believed that their own needs and opinions went unnoticed, and was reinforced in healthcare settings, both in relation to their child’s care and their own well-being.^32 43 51 52^ Some described a lack of recognition from professionals and felt that healthcare systems were predominantly biased towards engaging with mothers, leaving fathers on the periphery of their child’s care.^31–33 41 43 44 49 50 52 54 56 57 59–61^ This lack of interaction and communication resulted in fathers feeling helpless, surplus to requirement, isolated and out-of-control.^31 49 56 59 60^ Sometimes this exclusion was due to ‘competing responsibilities’^50^ though many fathers expressed a desire to be more involved in their child’s care.

Well I don’t know that they necessarily cared about me that much. I think that they were more concerned…about my wife be-cause she was the one that was carrying the child and having to deal with the issues. You know, they let me know what was basically going on but I was kind of odd man out.^56^ (quote from study participant, genetic conditions, USA)

Fathers experienced intense emotion; however, they described themselves as “alone,” “strong,” and “to themselves”.^44^ (quote from study authors, cancer, Canada)

Fathers experienced uncertainty and fear when professionals did not communicate effectively or involve them in the decision-making.^33 47 49 58^ In contrast to this, feeling understood and recognised by professionals seemed to help fathers to cope with their child’s diagnosis. Regardless of the news they received, if information was clear, they felt a greater sense of control and part of a team in their child’s care emphasising the importance of fathers feeling listened to, understood and an integral part of their child’s care rather than an ‘observer’^49^ or ‘spectator’.^56^ Fathers valued straightforward, honest, knowledgeable, approachable and calm professionals.^31 35 36 45 47 58 61^

Fathers also feel safe and recognized as an important member of the family when health care professionals not only give their time but also stand by the fathers’ side and help them fight for their families’ right to get information and be together.^47^ (congenital heart defect, UK)

### Working fathers: role conflict

Many fathers felt that it was their responsibility to earn money for their families, with some describing the financial ‘worry’ or ‘burden’ as falling to them to address.^35 37^ This was in part discussed in relation to their assumptions about the paternal role,^32 35 37 50^ and in part, in relation to what worked best for individual families.^33^ For some working fathers, balancing their roles was manageable, and work provided a respite function and an opportunity to discuss other things.^42 43^ Furthermore, several fathers did take on the role of primary caregiver, sometimes attributed to flexible workplaces or self-employment.^41 45^ For others, finding the balance between home and work was overwhelming, making it difficult to focus at work and prompting job security fears^53 59^ Similarly, some described a lack of opportunity to spend time with their child and family due to work commitments,^46^ sometimes due to being tired, contributing to feelings of guilt and isolation.

Juggling home routines, hospital visits, and work responsibilities, some fathers experienced a decrease in workplace productivity and in several cases, employer sanction or dismissal resulted. 44 (cancer, Canada)

I’m mostly just there to play with [the ill child]…Sometimes I feel guilty if I have a bad day at work or something like that, and she wants to play, and I’m tired or stuff like that, I sort of feel guilty in the back of my mind. 59 (life-limiting conditions, USA)

Work commitments also meant that some fathers were not able to attend their child’s appointments, leaving them with a further sense of disconnect from their families and from their child’s care. This lack of involvement made building relationship with the healthcare professionals very difficult. This sense of isolation was highlighted when fathers were unable to discuss their situation at work.

Yet another father sadly revealed that he had not been able to attend any appointments with his child for more than a year. 56 (genetic conditions, USA)

I’m in sales. Do you think my customers want to hear “Well gee, my daughter’s got a brain tumor”? So I keep that inside–so out of my 200 customers, maybe 12 know about her.^36^ (cancer, USA)

Having supportive employers and colleagues who granted fathers flexible working schedules and time off when needed, appeared to make things easier for fathers, not only in terms of workplace productivity, but in being able to balance life in a way that worked for them, adding an element of control to an uncertain situation. Furthermore, it made work a more pleasant place where fathers could maintain some ‘normality’. The value of this in making them feel understood, valued and in control was evident in fathers’ accounts of both positive and negative experiences of workplace support:

I lost my job because…sometimes you have to go “oh my son is sick”; some don’t understand…It’s not all the time they [are] going to accept it and you need your job to be done and you are not there all the time …So finally I ended up losing my job and my wife also lost her job because of that. 59 (quote from study participant, life-limiting conditions, Canada)

Flexible work arrangements and general workplace support were key factors that helped these fathers adjust to increased caretaking demands.^31^ (quote from study authors, cancer, USA)

## Discussion

This qualitative synthesis highlights the challenges and emotions experienced by fathers of children with a life-limiting condition beginning with a great deal of uncertainty in the period leading up to their child’s diagnosis. This uncertainty was amplified by a lack of information from healthcare professionals and fathers described feeling fearful and helpless. Adjustment and acceptance followed diagnosis, though the extent to which this occurred varied between individuals. They discussed the life-changing nature of their child’s diagnosis, an event effecting all aspects of their lives from their everyday actions, to their relationships, spirituality, values and ambitions. Fathers described themselves as supportive figures who often put the needs of their partner and child/children before their own. This included concealing their own emotions out of fear that they would cause distress for others. The way in which professionals engaged with fathers further validated their own perceptions of needing to be a strong and protective figure. Working fathers struggled to balance their roles at home and at work, and a lack of workplace support caused further distress.

Illness uncertainty has been described as ‘the appraisal of illness and its treatment as ambiguous or unpredictable, or feelings of having insufficient information to cognitively organise the illness event’ and has been associated with psychological distress in parents of children with chronic conditions.^63^ Fathers in this study described their uncertainty, throughout diagnosis and treatment, and partly attributed this to a lack of information surrounding their child’s condition. The way in which fathers managed this uncertainty, through emotional suppression and problem-focused coping for example, is consistent with experiences reflected in wider parental literature, paternal research in neonatal intensive care unit (NICU) settings^11 64 65^ and literature on coping.^66^ Uncertainty is common in paediatric palliative care, and coupled with a loss of control, appears to dominate many families’ experiences.^67^ Although the way in which fathers discussed and managed their uncertainty related to their child’s condition did not differ noticeably to what is represented in existing literature, this review emphasises the extent to which it occupied their experiences, and highlights fathers’ unique perspectives of both contributing factors and means by which they felt empowered and able to adapt to uncertainty. Existing studies recommend targeting this uncertainty as a means of reducing psychological distress.^63^

Wider parental literature on childhood life-limiting illnesses describes parents as ‘travelling a different pathway’ to the expected.^16^ For many parents, life becomes about ‘battling’ their child’s illness, and day-day-day living revolves around such.^18 68^ Shifts in existing relationships with friends due to a loss of common interests can lead to a withdrawal from existing social circles and a shift towards those with a shared understanding.^69^

Fathers’ experiences of grief, shock, devastation and sadness are also synonymous with those described in wider maternal and parental palliative care literature.^9 11 70^ However, this review emphasised how fathers’ experiences were shaped by gendered ideas of emotional expression, with their minimal outward display of emotion satisfying the ‘strong and stoic’ traditional sociocultural idea of masculinity.^71^ Some explicitly linked their reluctance to discuss their emotions to masculinity, while for others it was linked to a more subliminal desire to protect their families from further emotional distress. Not so clear, were the differences between fathers for whom performing this supportive role was helpful and for whom it was fulfilled out of perceived obligation, though many fathers described a helplessness leading to role uncertainty.

This role uncertainty has been described in wider literature on fathers’ transitions to parenthood.^72^ In the context of life-limiting illness, this element of role uncertainty is a particular struggle given that fathers feel unable to protect their child against their condition. This issue was reflected in the way in which fathers described their experiences in healthcare settings, sometimes feeling a lack of involvement often due to a perceived lack of engagement from healthcare professionals. The issue of father involvement and support is reflected across paediatric healthcare settings and is improving, particularly in NICU settings.^65^ Fathers have been shown to seek help, participate in decision-making and contribute to their child’s care when they feel accepted and comfortable enough to be assertive, though engaging them can be difficult if they already perceive their role to be a supportive one.^73^

Closely related to this are the challenges faced by working fathers in balancing responsibilities at home and at work. There were few fathers in this study who assumed the role of primary caregiver for their child, with the majority being in employment, following the traditional role division that still exists more generally.^13^ However, many fathers expressed a desire to be more heavily involved in their child’s care, which was sometimes made difficult by their workplace responsibilities. Studies show that employees with caregiving responsibilities are at a higher risk of negative workplace outcomes, such as dismissal^74 75^ which was reflected in fathers’ anxieties related to their performance at work. Research also shows that limited uptake of additional paternity leave is in part due to concerns over a lack of employer support^76^ which further highlights the struggles faced more generally by fathers at work. Existing research focuses heavily on female caregiver discrimination in the workplace,^77^ meaning that not much is known about problems faced by men when they have caregiving responsibilities, particularly fathers of unwell children for whom treatment and caring responsibilities may extend over many years. On the other hand, flexible working policies have increased across the workforce in recent years^78^ and allow for individuals to balance their work–home roles more easily which was also reflected in fathers’ positive accounts of workplace support. However, access to flexible working arrangements are not uniform and are affected by occupational group.^78^ Other positive experiences related to fathers’ work serving as a respite function, allowing them to maintain a sense of self. These positive and negative experiences emphasise the importance of workplace support for parents and the recognition that more fathers want to be involved in the care of their children as well as maintain employment.

## Strengths and limitations of included studies

This review had a number of strengths. It included the experiences of 576 fathers across multiple countries and settings. Findings were drawn from these perspectives, shedding light on the unique experiences of fathers. Thematic synthesis allowed for rigorous analysis and the inclusion of a studies from a range of methodologies, settings and diagnoses. As far as we aware, this is the first review in this area that includes father-only studies. A recent meta-ethnography of fathers’ experiences of caring for a child with a life-limiting condition, included studies in which mothers’ experiences were also sought.^79^ Including ‘father-only’ studies was a significant criterion in protocol development, as discussed with a family advisory board of parents of children with life-limiting conditions, who felt that this was important. Many of our findings share similarities with those of Postavaru and Swaby^79^ emphasising the uncertainty, helplessness and isolation often experienced by these fathers, as well as the impact of the perception of the male role in shaping these experiences. In this review, this was emphasised through the role conflict experienced by working fathers.

It was beyond the scope of the review to include studies published in languages other than English. Fifteen of the included studies were focused on the experiences of fathers of children with cancer and those that included a broad range of life-limiting conditions also included fathers of children with cancer, meaning that there was further bias towards oncology settings. Studies were published internationally and reflected the views of fathers across an array of healthcare structures. Studies were published between 1997 and 2019 and data were collected between 1978 and 2019 providing experiences that span many years, across changing healthcare systems, medical developments and societal views. There was a lack of cultural diversity among the included participants. Inconsistent indexing across databases, as well as use of non-standardised terminology across qualitative methodologies and low quality or absence of abstracts, contributes to the complexities of locating studies in qualitative research. However, the search strategy was extensive and allowed for the identification of 32 papers from 30 studies.

## Conclusion

This review explored the experiences of fathers of children with a life-limiting condition. Four main themes were identified: fathers’ experiences of uncertainty, a ‘new life’, their reluctance to discuss emotions and how this can be reinforced by interactions with healthcare professionals and some of the challenges faced by working fathers. Father’s described their experiences of grief, trauma, shock, devastation, exhaustion, hypervigilance and uncertainty, though few studies explored well-being specifically and the longer-terms effects of such are unknown. In addition to these difficulties, fathers often faced problems in forming relationships with healthcare professionals and role conflict in trying to maintain employment throughout their child’s illness, contributing to them feeling like observers in their child’s care. Workplaces should strive for flexible policy that allows for both mothers and fathers to be involved in their child’s ongoing care, especially as parental roles are evolving beyond the more dichotomous traditional norms. Further research is required to understand the nuances of working fathers’ work and home role balance and what this means in terms of support needs.

Many of the issues discussed by fathers are risk factors for poor psychological outcomes, but further research is needed to assess the burden of such on fathers of children with a life-limiting condition. Furthermore, research is heavily situated in oncology settings meaning that parents of children with other conditions are under-represented. These studies demonstrate that although historically it has been difficult to recruit fathers, it is possible to do so in adequate numbers. However, the recruitment of fathers in future research should aim for more diversity; culturally, in diagnostic groups and in recruiting fathers from different family structures.

10.1136/bmjspcare-2021-003019.supp3 Supplementary data

## Data Availability

Data sharing not applicable as no datasets generated and/or analysed for this study.
