# Supplementary material for: Experiences of fathers of children with a life-limiting condition: a systematic review and qualitative synthesis
Source: BMJ Support Palliat Care. 2021 Jun 17;13(1):15–26. doi: 10.1136/bmjspcare-2021-003019 (PMC9985706; doi:10.1136/bmjspcare-2021-003019)
Supplement: Supplementary data [file bmjspcare-2021-003019supp001.pdf]

Supplemental material; search strategy for Medline

1. Fathers/
2. father*.tw.
3. dad*.tw.
4. stepfather*.tw.
5. step-father*.tw.
6. (foster* adj3 father*).tw.
7. (adopt* adj3 father*).tw.
8. 1 or 2 or 3 or 4 or 5 or 6 or 7
9. child/ or child, preschool/ or infant/
10. Adolescent/
11. (child$ or children$).tw.
12. (infant$ or infancy$).tw.
13. (baby or baby$ or babies).tw.
14. (toddler$ or kid or kids).tw.
15. (boy or boys or boyhood or girl or girls or girlhood).tw.
16. (minor or minor$ or schoolchild$).tw.
17. (adolescen$ or juvenil$ or youth$ or teen$ or "under age$" or underage$ or pubescen$).tw.
18. (pediatric$ or paediatric$ or peadiatric$).tw.
19. (young people$ or young person$).tw.
20. young adult$.tw.
21. 9 or 10 or 11 or 12 or 13 or 14 or 15 or 16 or 17 or 18 or 19
22. life-limiting illness*.tw.
23. life-limiting condition*.tw.
24. life-limiting disease*.tw.
25. life-threatening illness*.tw.
26. life-threatening condition*.tw.
27. life-threatening disease*.tw.
28. Creutzfeldt-Jakob Syndrome/
29. (creutzfeldt-jakob$ or jakob-creutzfeldt$ or cjd or spongiform encephalopath$).ti,ab,kf.
30. Subacute Sclerosing Panencephalitis/
31. (subacute sclerosing panencephalit$ or sub-acute sclerosing panencephalit$ or sspe or subacute sclerosing leukoencephalit$ or sub-acute sclerosing leukoencephalit$ or van bogaert$ leukoencephalit$ or measles inclusion body encephalit$ or mibe).ti,ab,kf.
32. beta-Thalassemia/
33. (beta adj (thalass?emi$ or thalas?emi$)).ti,ab,kf.
34. ((thalass?emi$ or thalas?emi$) adj major).ti,ab,kf.
35. exp Anemia, Aplastic/
36. ((hypoplastic or aplastic) adj an?emi$).ti,ab,kf.
37. (medullary adj3 hypoplas$).ti,ab,kf.
38. exp Neutropenia/
39. ((severe or chronic$) adj3 neutropeni$).ti,ab,kf.

40. immunologic deficiency syndromes/ or acquired immunodeficiency syndrome/
41. (immun$ deficiency adj (syndrome$ or disease$ or disorder$)).ti,ab,kf.
42. (immunodeficiency adj (syndrome$ or disease$ or disorder$)).ti,ab,kf.
43. DiGeorge Syndrome/
44. (digeorge$ or di george$ or sedlackova$ or opitz g-bbb or velocardiofacial or velo-cardiofacial or velo-cardio-facial or shprintzen$ or ctaf).ti,ab,kf.
45. ((deletion or vcf or pharyngeal pouch or thymic aplasia or anomaly face) adj (syndrome$ or disease$ or disorder$)).ti,ab,kf.
46. Common Variable Immunodeficiency/
47. ((common variable or late onset) adj3 (immunodeficienc$ or immune deficienc$ or immunoglobulin deficienc$ or hypogammaglobulin$)).ti,ab,kf.
48. acquired hypogammaglobulin$.ti,ab,kf.
49. Cryoglobulinemia/
50. cryoglobulin?em$.ti,ab,kf.
51. Polyendocrinopathies, Autoimmune/
52. ((autoimmune or failure$) adj3 (polyglandular$ or polyendocrin$)).ti,ab,kf.
53. Progeria/
54. (progeria or hutchinson-gilford$).ti,ab,kf.
55. Tyrosinemias/
56. tyrosin?em$.ti,ab,kf.
57. Maple Syrup Urine Disease/
58. (maple syrup urine or msud).ti,ab,kf.
59. branched chain.ti,ab,kf.
60. (bckd adj5 (deficienc$ or ketoacid$ or keto-acid$)).ti,ab,kf.
61. hyperleucine-isoleucin$.ti,ab,kf.
62. Methylmalonic Acid/
63. (methylmalonic acid?emi$ or methylmalonic aciduri$ or methyl malonic acid?emi$ or methyl malonic aciduri$).ti,ab,kf.
64. Propionic Acidemia/
65. (propionic acid?em$ or propionic acidur$ or propionyl-CoA carboxylase deficienc$ or ketotic glycin?em$).ti,ab,kf.
66. Adrenoleukodystrophy/
67. (adrenoleukodystroph$ or x-ald or schilder-addison$ or addison-schilder$ or adrenomyeloneuropath$).ti,ab,kf.
68. Carnitine O-Palmitoyltransferase/
69. ((carnitine palmityltransferase or carnitine palmitoyltransferase or carnitine o-palmityltransferase or carnitine o-palmitoyltransferase) adj3 deficienc$).ti,ab,kf.
70. Fanconi Syndrome/
71. (fanconi$ adj (syndrome$ or disease$ or disorder$)).ti,ab,kf.
72. Cystinosis/
73. (cystinos$ or cystine storage or cystine diathes$ or cystine disease$).ti,ab,kf.
74. Oculocerebrorenal Syndrome/
75. ((lowe or lowes or oculocerebrorenal or cerebrooculorenal or cerebro-oculo-renal) adj3 (syndrome$ or disease$ or disorder$)).ti,ab,kf.
76. Metalloproteins/df [Deficiency]
77. Molybdenum/df [Deficiency]

78. (molybdenum cofactor deficien$ or molybdenum co-factor deficien$).ti,ab,kf.
79. Oxidoreductases Acting on Sulfur Group Donors/df [Deficiency]
80. Sulfite Oxidase/df [Deficiency]
81. ((sulphite$ or sulfite$) adj3 oxidase deficien$).ti,ab,kf.
82. Argininosuccinic Acid/
83. (argininosuccinic acidur$ or argininosuccinic acid?emi$).ti,ab,kf.
84. Citrullinemia/
85. (citrullin?emi$ or citrullinuri$).ti,ab,kf.
86. Amino Acid Metabolism, Inborn Errors/
87. (glutaric acid?emi$ or glutaric aciduri$).ti,ab,kf.
88. Hyperglycinemia, Nonketotic/
89. (glycine encephalopath$ or non-ketotic hyperglycin?emi$ or nonketotic hyperglycin?emi$).ti,ab,kf.
90. Hyperargininemia/
91. (arginin?emi$ or arginase deficien$ or hyperarginin?emi$).ti,ab,kf.
92. Renal Aminoacidurias/
93. (aminoaciduri$ or aminoacid?emi$).ti,ab,kf.
94. exp glycogen storage disease/
95. (glycogen storage adj (disease$ or syndrome$ or disorder$)).ti,ab,kf.
96. (pompe$ adj (disease$ or syndrome$ or disorder$)).ti,ab,kf.
97. Galactosemias/
98. galactos?emi$.ti,ab,kf.
99. Pyruvate Dehydrogenase Complex Deficiency Disease/
100. (pyruvate dehydrogenase adj3 deficien$).ti,ab,kf.
101. (oxalosis and (renal or kidney$)).ti,ab,kf.
102. exp Gangliosidoses/
103. gangliosidos$.ti,ab,kf.
104. (sandhoff$ adj (disease$ or syndrome$ or disorder$)).ti,ab,kf.
105. tay sach$.ti,ab,kf.
106. Mucolipidoses/
107. mucolipidos$.ti,ab,kf.
108. Canavan Disease/
109. (canavan$ leucodystroph$ or aspartoacylase deficien$ or aminoacylase 2 deficien$).ti,ab,kf.
110. ((canavan$ or canavan-van bogaert-bertrand$) adj (disease$ or syndrome$ or disorder$)).ti,ab,kf.
111. Gaucher Disease/
112. (gaucher$ adj (disease$ or syndrome$ or disorder$)).ti,ab,kf.
113. (glucocerebrosidase deficien$ or glucosylceramidase deficien$).ti,ab,kf.
114. Leukodystrophy, Metachromatic/
115. (metachromatic leukodystroph$ or arylsulfatase A deficien$ or metachromic leukodystroph$).ti,ab,kf.
116. exp Niemann-Pick Diseases/
117. (niemann-pick$ or sphingomyelinase deficien$).ti,ab,kf.

118. Sphingolipidoses/
119. sphingolipidos$.ti,ab,kf.
120. Fabry Disease/
121. (fabry$ adj (disease$ or syndrome$ or disorder$)).ti,ab,kf.
122. (angiokeratoma corporis diffusum or alpha-galactosidase A deficien$).ti,ab,kf.
123. Leukodystrophy, Globoid Cell/
124. (krabbe$ adj (disease$ or syndrome$ or disorder$)).ti,ab,kf.
125. (globoid cell leukodystroph$ or galactosylceramide lipidos$ or galactosylcerebrosidase deficien$ or galactosylceramidase deficien$).ti,ab,kf.
126. Farber Lipogranulomatosis/
127. (farber$ adj (disease$ or syndrome$ or disorder$)).ti,ab,kf.
128. (farber$ lipogranulomatos$ or ceramidase deficien$ or fibrocytic dysmucopolysaccharidos$).ti,ab,kf.
129. Pelizaeus-Merzbacher Disease/
130. pelizaeus-merzbacher$.ti,ab,kf.
131. Sulfatases/df [Deficiency]
132. Multiple Sulfatase Deficiency Disease/
133. (sulfatase deficien$ or sulphatase deficien$ or mucosulfatidos$).ti,ab,kf.
134. (austin$ adj (disease$ or syndrome$ or disorder$)).ti,ab,kf.
135. sulfatidos$.ti,ab,kf.
136. Sea-Blue Histiocyte Syndrome/
137. sea-blue histiocyt$.ti,ab,kf.
138. Neuronal Ceroid-Lipofuscinoses/
139. (batten$ adj (disease$ or syndrome$ or disorder$)).ti,ab,kf.
140. (neuronal ceroid lipofuscinos$ or santavuori-haltia$ or jansky-bielschowsky$ or bielschowsky-jansky$).ti,ab,kf.
141. (kuf$ adj (disease$ or syndrome$ or disorder$)).ti,ab,kf.
142. spielmeyer vogt$.ti,ab,kf.
143. Xanthomatosis, Cerebrotendinous/
144. ((cerebrotendineous or cerebrotendinous or cerebrotendious or cerebral) adj3 (xanthomatos$ or cholesteros$)).ti,ab,kf.
145. bogaert-scherer-epstein$.ti,ab,kf.
146. Wolman Disease/
147. (wolman$ adj (disease$ or syndrome$ or disorder$)).ti,ab,kf.
148. lysosomal acid lipase deficien$.ti,ab,kf.
149. exp Mucopolysaccharidoses/
150. mucopolysaccharidos$.ti,ab,kf.
151. (hurler$ adj2 (syndrome$ or disease$ or disorder$)).ti,ab,kf.
152. (hunter$ adj2 (syndrome$ or disease$ or disorder$)).ti,ab,kf.
153. (MPS1 or MPS2 or MPS3 or MPS4 or MPS5 or MPS6 or MPS7 or MPS-1 or MPS-2 or MPS-3 or MPS-4 or MPS-5 or MPS-6 or MPS-7 or MPSI or MPSII or MPSIII or MPSIV or MPSV or MPSVI or MPSVII or MPS-I or MPS-II or MPS-III or MPS-IV or MPS-V or MPS-VI or MPS-VII).ti,ab,kf.
154. (beta glucuronidase deficien$ or sly syndrome$ or sly disorder$ or sly disease$).ti,ab,kf.
155. (maroteaux-lamy$ or marotaeux-lamy$ or polydystrophic dwarfism).ti,ab,kf.

156. (morquio$ or moriquio$ or beta galactosidase deficien$).ti,ab,kf.
157. (sanfilippo$ or sanfillipo$).ti,ab,kf.
158. Mucolipidoses/
159. (mucolipidos$ or pseudo-hurler$ or pseudohurler$).ti,ab,kf.
160. ((inclusion-cell or i-cell) adj (disease$ or syndrome$ or disorder$)).ti,ab,kf.
161. Fucosidosis/
162. (fucosidos$ or fucidos$).ti,ab,kf.
163. "Congenital Disorders of Glycosylation"/
164. ((cdg or ctg) adj (disease$ or disorder$ or syndrome$)).ti,ab,kf.
165. (carbohydrate-deficient glycoprotein adj (disease$ or disorder$ or syndrome$)).ti,ab,kf.
166. (congenital disorder$ adj3 glycosylation).ti,ab,kf.
167. Lesch-Nyhan Syndrome/
168. ((nyhan$ or kelley-seegmiller$) adj (syndrome$ or disorder$ or disease$)).ti,ab,kf.
169. juvenile gout.ti,ab,kf.
170. Menkes Kinky Hair Syndrome/
171. menkes$.ti,ab,kf.
172. ((copper transport or steely hair or kinky hair) adj (disease$ or syndrome$ or disorder$)).ti,ab,kf.
173. alpha 1-Antitrypsin Deficiency/
174. (antitrypsin deficien$ or A1AD).ti,ab,kf.
175. (AAT deficien$ or alpha-1 protease deficien$).ti,ab,kf.
176. bisalbumin?emi$.ti,ab,kf.
177. Lipodystrophy, Congenital Generalized/
178. (congenital generali?ed lipodystroph$ or berardinelli$ or bernardnelli$).ti,ab,kf.
179. Landau-Kleffner Syndrome/
180. (landau-kleffner$ or infantile acquired aphasia$ or acquired epileptic aphasia$).ti,ab,kf.
181. (aphasia$ adj5 convulsive).ti,ab,kf.
182. Rett Syndrome/
183. (rett$ adj (syndrome$ or disease$ or disorder$)).ti,ab,kf.
184. cerebroatrophic hyperammon?emi$.ti,ab,kf.
185. Huntington Disease/
186. huntington$.ti,ab,kf.
187. exp Spinocerebellar Ataxias/
188. (spinocerebellar ataxia$ or ataxia$ telangiectasia$ or louis-bar$ syndrome$ or louis-bar$ disease$ or louis-bar$ disorder$ or machado-joseph$ or joseph$ disease$ or joseph$ disorder$ or joseph$ syndrome$).ti,ab,kf.
189. Friedreich Ataxia/
190. ((friedreich$ or friedrich$) adj3 ataxia$).ti,ab,kf.
191. spinocerebellar degenerat$.ti,ab,kf.
192. "Spinal Muscular Atrophies of Childhood"/
193. (spinal muscular atroph$ or werdnig hoffman$).ti,ab,kf.
194. (dubowitz$ or kugelberg-welander$).ti,ab,kf.
195. Bulbar Palsy, Progressive/
196. (fazio-londe$ or faziolonde$ or progressive bulbar pals$).ti,ab,kf.

197. Pantothenate Kinase-Associated Neurodegeneration/
198. (pantothenate kinase-associated neurodegenerat$ or PKAN or hallervorden-spatz$).ti,ab,kf.
199. ((neurodegeneration adj3 brain iron accumulation) or NBIA$1).ti,ab,kf.
200. Olivopontocerebellar Atrophies/
201. (olivopontocerebellar atroph$ or OPCA or olivopontocerebellar degenerat$).ti,ab,kf.
202. (multiple system atrophy adj5 cerebellar).ti,ab,kf.
203. "Diffuse Cerebral Sclerosis of Schilder"/
204. (alper$ adj (disease$ or syndrome$ or disorder$)).ti,ab,kf.
205. (progressive sclerosing poliodystroph$ or progressive infantile poliodystroph$).ti,ab,kf.
206. (diffuse cerebral sclerosis adj5 schilder$).ti,ab,kf.
207. Leigh Disease/
208. (leigh$ adj (syndrome$ or disease$ or disorder$)).ti,ab,kf.
209. (subacute necrotizing encephalomyelopath$ or subacute necrotising encephalomyelopath$ or sub-acute necrotizing encephalomyelopath$ or sub-acute necrotising encephalomyelopath$ or SNEM).ti,ab,kf.
210. (aicardi-gouti?res or aicardia-gouti?res).ti,ab,kf.
211. (worster-drought$ or congenital suprabulbar pares$).ti,ab,kf.
212. multiple sclerosis/ or multiple sclerosis, chronic progressive/ or multiple sclerosis, relapsing-remitting.mp.
213. (multiple sclerosis or disseminated sclerosis or encephalomyelitis disseminata$).ti,ab,kf.
214. (demyelinating adj (disease$ or syndrome$ or disorder$)).ti,ab,kf.
215. exp Epilepsies, Myoclonic/
216. myoclonic epileps$.ti,ab,kf.
217. ((lafora$ or merrf$ or unverricht-lundborg$ or janz$) adj (disease$ or syndrome$ or disorder$)).ti,ab,kf.
218. lennox-gastaut$.ti,ab,kf.
219. (lennox$ adj (syndrome$ or disease$ or disorder$)).ti,ab,kf.
220. Spasms, Infantile/
221. (west$ adj (syndrome$ or disease$ or disorder$)).ti,ab,kf.
222. Epilepsia Partialis Continua/
223. (epilepsia partialis continua or kojevnikov$ or epilepsia partialis continuoa or kozhevnikof$).ti,ab,kf.
224. Charcot-Marie-Tooth Disease/
225. (charcot-marie-tooth$ or peroneal muscular atroph$).ti,ab,kf.
226. (progressive neuropathic muscular atroph$ or hereditary peroneal nerve dysfunction$ or peroneal neuropath$).ti,ab,kf.
227. "Hereditary Sensory and Motor Neuropathy"/
228. (hereditary sensory adj3 motor neuropath$).ti,ab,kf.
229. (hereditary motor adj3 sensory neuropath$).ti,ab,kf.
230. Refsum Disease, Infantile/
231. Peroxisomal Disorders/
232. (infantile refsum or infantile phytanic acid storage).ti,ab,kf.
233. Myasthenic Syndromes, Congenital/
234. congenital myasth?eni$.ti,ab,kf.

235. Muscular Dystrophy, Duchenne/
236. (duchenne muscular dystroph$ or dmd).ti,ab,kf.
237. exp Muscular Dystrophies, Limb-Girdle/
238. (limb-girdle or erb$ muscular dystroph$).ti,ab,kf.
239. (sarcoglycanopath$ or sarcoglycaopath$).ti,ab,kf.
240. Osteochondrodysplasias/
241. (osteochondrodysplas$ or schwartz-jampel or chondrodystrophi$ myotoni$ or myotoni$ chondrodystrophi$).ti,ab,kf.
242. Myotonia Congenita/
243. (congenita$ myotoni$ or myotoni$ congenita$).ti,ab,kf.
244. (thomsen$ adj (disease$ or disorder$ or syndrome$)).ti,ab,kf.
245. ((recessive adj3 myotoni$) or becker$ myotoni$).ti,ab,kf.
246. Isaacs Syndrome/
247. (isaac$ adj (syndrome$ or disease$ or disorder$)).ti,ab,kf.
248. neuromyotoni$.ti,ab,kf.
249. Myotonic Disorders/
250. (paramyotoni$ congenita$ or congenita$ paramyotoni$).ti,ab,kf.
251. (eulenburg$ adj (disease$ or syndrome$ or disorder$)).ti,ab,kf.
252. (myotoni$ adj (disease$ or disorder$ or syndrome$)).ti,ab,kf.
253. pseudomyotoni$.ti,ab,kf.
254. exp Myopathies, Structural, Congenital/
255. (congenital adj3 myopath$).ti,ab,kf.
256. myopathycongenital.ti,ab,kf.
257. ((nemaline or rod) adj3 myopath$).ti,ab,kf.
258. ((central core or mini-core or minicore or multicore or multi-core) adj (disease$ or disorder$ or syndrome$ or myopath$)).ti,ab,kf.
259. fiber type disproportion.ti,ab,kf.
260. fibre type disproportion.ti,ab,kf.
261. Muscular Dystrophies/cn [Congenital]
262. (congenital$ adj5 muscular dystroph$).ti,ab,kf.
263. ((centronuclear or myotubular) adj myopath$).ti,ab,kf.
264. exp Mitochondrial Myopathies/
265. (mitochondrial myopath$ or mitochondrial encephalomyopath$ or chronic progressive external ophthalmopleg$).ti,ab,kf.
266. ((melas or kearns-sayre$) adj (syndrome$ or disease$ or disorder$)).ti,ab,kf.
267. Quadriplegia/ and spastic$.ti,ab,kf.
268. (spastic quadriplegi$ or spastic tetraplegi$).ti,ab,kf.
269. Reye Syndrome/
270. (reye$ adj (syndrome$ or disease$ or disorder$)).ti,ab,kf.
271. multiple pterygium.ti,ab,kf.
272. Hypertension, Pulmonary/ and primary$.ti,ab,kf.
273. ((primary pulmonary or precapillary pulmonary or idiopathic pulmonary) adj (hypertension or ht or arterial hypertension)).ti,ab,kf.
274. ((primary bronchopulmonary or precapillary bronchopulmonary or idiopathic bronchopulmonary) adj (hypertension or ht or arterial hypertension)).ti,ab,kf.

275. ((primary lung or precapillary lung or idiopathic lung) adj (hypertension or ht or arterial hypertension)).ti,ab,kf.
276. ipah.ti,ab,kf.
277. Cardiomyopathy, Dilated/
278. ((congestive or dilated) adj cardiomyopath$).ti,ab,kf.
279. exp Cardiomyopathy, Hypertrophic/
280. (hypertrophic adj cardiomyopath$).ti,ab,kf.
281. Cardiomyopathies/cn [Congenital]
282. (congenital adj3 cardiomyopath$).ti,ab,kf.
283. Cardiomyopathy, Restrictive/
284. (restrictive cardiomyopath$ or obliterative cardiomyopath$ or constrictive cardiomyopath$).ti,ab,kf.
285. exp Pulmonary Fibrosis/
286. (pulmonary fibros$ or lung fibros$ or bronchopulmonary fibros$ or fibrosing alveolit$ or interstitial pneumonit$).ti,ab,kf.
287. Respiratory Insufficiency/
288. (respiratory adj (failure$ or insufficienc$)).ti,ab,kf.
289. "Cystic Adenomatoid Malformation of Lung, Congenital"/
290. ((cystic lung or cystic pulmonary or cystic bronchopulmonary) adj (disease$ or disorder or syndrome$)).ti,ab,kf.
291. (bronchogenic cyst$ or bronchopulmonary foregut malformation$).ti,ab,kf.
292. cystic adenomatoid malformation$.ti,ab,kf.
293. lobar emphysem$.ti,ab,kf.
294. (pulmonary sequestration$ or bronchopulmonary sequestration$ or lung sequestration$ or extralobar sequestration$ or extra-lobar sequestration$ or intralobar sequestration$ or intra-lobar sequestration$).ti,ab,kf.
295. pulmolithias$.ti,ab,kf.
296. exp Liver Failure/
297. ((liver$1 or hepatic) adj3 fail$).ti,ab,kf.
298. exp Liver Cirrhosis/
299. (cirrhosis adj3 liver$1).ti,ab,kf.
300. Hepatic Veno-Occlusive Disease/
301. ((veno-occlusive or venous occlusive) adj (disease$ or syndrome$ or disorder$)).ti,ab,kf.
302. Exocrine Pancreatic Insufficiency/
303. (swachman-diamond or shwachman-bodian or schwachmann-diamond or shwachmann-bodian).ti,ab,kf.
304. Granulomatosis with Polyangiitis/
305. wegener$ granulomatos$.ti,ab,kf.
306. (granulomatos$ adj3 polyangiit$).ti,ab,kf.
307. Osteolysis, Essential/
308. essential osteolys$.ti,ab,kf.
309. ((gorham$ or gorham-stout$ or vanishing bone or phantom bone) adj (disease$ or syndrome$ or disorder)).ti,ab,kf.
310. ((arc or arthrogryposis renal dysfunction cholestasis) adj (disease$ or syndrome$ or disorder)).ti,ab,kf.
311. Cerebral Hemorrhage/cn [Congenital]

312. Cerebral Hemorrhage, Traumatic/
313. Cerebral Hemorrhage/ and Birth Injuries.mp.
314. (cerebral h?emorrhage$ and (birth$ adj3 injur$)).ti,ab,kf.
315. Asphyxia Neonatorum/
316. asphyxia neonatorum.ti,ab,kf.
317. ((perinatal$ or neonatal$ or birth$) adj3 asphyxia$).ti,ab,kf.
318. Rubella Syndrome, Congenital/
319. congenital rubella.ti,ab,kf.
320. exp Cytomegalovirus Infections/cn [Congenital]
321. (congenital adj (cytomegalovirus$ or cmv)).ti,ab,kf.
322. Chickenpox/cn [Congenital]
323. exp Herpes Zoster/cn [Congenital]
324. Herpesvirus 3, Human/ and congenital$.ti,ab,kf.
325. ((congenital or fetal or foetal) adj3 (varicella$ or chicken pox$ or VZV)).ti,ab,kf.
326. Toxoplasmosis, Congenital/
327. congenital toxoplasmos$.ti,ab,kf.
328. exp Hypoxia, Brain/
329. ((brain$ or cerebral) adj3 hypoxi$).ti,ab,kf.
330. Renal Insufficiency/cn [Congenital]
331. Acute Kidney Injury/cn [Congenital]
332. Renal Insufficiency, Chronic/cn [Congenital]
333. Kidney Failure, Chronic/cn [Congenital]
334. (congenital$ adj3 (kidney failure$ or renal failure$ or kidney insufficienc$ or renal insufficienc$)).ti,ab,kf.
335. (congenital$ adj3 (kidney disease$ or renal disease$)).ti,ab,kf.
336. Anencephaly/
337. (anencephal$ or meroanencephal$ or craniorachischis$).ti,ab,kf.
338. (aprosencephal$ adj3 open cranium).ti,ab,kf.
339. Encephalocele/
340. (encephalocele$ or cranium bifidum).ti,ab,kf.
341. Dandy-Walker Syndrome/
342. dandy-walker$.ti,ab,kf.
343. Acrocallosal Syndrome/
344. (acrocallosal or acro-callosal or acrocolossal or acro colossal).ti,ab,kf.
345. Aicardi Syndrome/
346. (aicardi$ adj (syndrome$ or disease$ or disorder$)).ti,ab,kf.
347. Holoprosencephaly/
348. (holoprosencephal$ or arhinencephal$ or holosprosencephal$).ti,ab,kf.
349. Hydranencephaly/
350. (hydranencephal$ or hydrancephal$ or hydroanencephal$).ti,ab,kf.
351. exp Lissencephaly/
352. Microcephaly/
353. (lissencephal$ or walker-warburg$ or miller-dieker$ or norman-robert$ or microlissencephal$).ti,ab,kf.

354. ((fukuyama$ or muscle-eye-brain) adj (syndrome$ or disease$ or disorder$)).ti,ab,kf.
355. "Malformations of Cortical Development"/
356. (microgyria$ or microgyrus or micro-gyria$ or micro-gyrus).ti,ab,kf.
357. (pachygyria$ or pachgyria$).ti,ab,kf.
358. agyria$.ti,ab,kf.
359. Septo-Optic Dysplasia/
360. ((septo-optic or septooptic) adj dysplas$).ti,ab,kf.
361. de morsier$.ti,ab,kf.
362. (schizencephal$ or schizzencephal$).ti,ab,kf.
363. Arnold-Chiari Malformation/
364. chiari$ malformation$.ti,ab,kf.
365. Truncus Arteriosus, Persistent/
366. (truncus or common arterial trunk$).ti,ab,kf.
367. "Transposition of Great Vessels"/
368. ((transposition$ or dextrotransposition$ or dtransposition$ or levotransposition$ or ltransposition$) adj3 (great arter$ or main arter$ or aorta$ or pulmonary arter$ or great vessel$ or main vessel$)).ti,ab,kf.
369. (dextro-tga or d-tga or levo-tga or l-tga).ti,ab,kf.
370. (double inlet adj3 ventricle$).ti,ab,kf.
371. DILV.ti,ab,kf.
372. single ventricle$.ti,ab,kf.

373. Heart Defects, Congenital/ and Atrial Appendage.mp.
374. (isomerism adj3 atrial appendage$).ti,ab,kf.
375. (aspleni$ or polyspleni$ or poly-spleni$).ti,ab,kf.
376. "Tetralogy of Fallot"/
377. (tetralogy adj3 fallot$).ti,ab,kf.
378. Eisenmenger Complex/
379. (eisenmenger$ or tardive cyanos$ or eisenmeyer$).ti,ab,kf.
380. (pentalogy adj3 fallot$).ti,ab,kf.
381. Pulmonary Atresia/
382. ((pulmonary or bronchopulmonary or lung$) adj3 atresia$).ti,ab,kf.
383. Tricuspid Atresia/
384. ((tricuspid or tri) adj3 atresia$).ti,ab,kf.
385. Ebstein Anomaly/
386. (ebstein$ adj (anomal$ or malformation$)).ti,ab,kf.
387. Hypoplastic Left Heart Syndrome/
388. (hypoplastic left heart adj (syndrome$ or disease$ or disorder$)).ti,ab,kf.
389. ((aortic or aorta$) adj3 atresia$).ti,ab,kf.
390. (mitral adj3 atresia$).ti,ab,kf.
391. ((absence$ or absent$) adj3 (aorta$ or aortic)).ti,ab,kf.
392. (aplas$ adj3 (aorta$ or aortic)).ti,ab,kf.
393. exp Aortic Aneurysm/cn [Congenital]
394. (((aorta$ or aortic) adj3 aneurys$) and congenital$).ti,ab,kf.

395. (hypoplas$ adj3 (aorta$ or aortic)).ti,ab,kf.
396. (convulsion$ adj3 (aorta$ or aortic)).ti,ab,kf.
397. (persistent right adj3 (aorta$ or aortic)).ti,ab,kf.
398. ((anomalous pulmonary venous or anamolous pulmonary venous) adj (connection or drainage or return)).ti,ab,kf.
399. ((absence$ or absent$) adj3 vena$ cava$).ti,ab,kf.
400. (persistent left adj3 cardinal vein$).ti,ab,kf.
401. Scimitar Syndrome/
402. ((scimitar$ or pulmonary venolobar) adj (syndrome$ or disease$ or disorder$)).ti,ab,kf.
403. (arteriovenous malformations/ or intracranial arteriovenous malformations/) and bilateral.ti,ab,kf.
404. ((bilateral AV or bilateral arteriovenous or bilateral arterio-venous) adj3 malform$).ti,ab,kf.
405. ((trachea$ or windpipe$ or wind-pipe$) adj3 atresia$).ti,ab,kf.
406. Tracheal Stenosis/
407. ((trachea$ or laryngotrachea$ or glottic or subglottic or sub-glottic) adj3 stenosis).ti,ab,kf.
408. Bronchopulmonary Dysplasia/
409. ((lung$ or pulmonary or bronchopulmonary) adj3 (hypoplas$ or dysplas$)).ti,ab,kf.
410. ((absence$ or absent$) adj3 (esophag$ or oesophag$ or foodpipe or food-pipe$ or gullet$)).ti,ab,kf.
411. Intestinal Atresia/
412. (duoden$ adj3 atresia$).ti,ab,kf.
413. ((absence$ or absent$) adj3 (intestin$ or gastrointestin$)).ti,ab,kf.
414. ((intestin$ or gastrointestin$) adj3 atresia$).ti,ab,kf.
415. ((intestin$ or gastrointestin$) adj3 stenos$).ti,ab,kf.
416. (cloaca$ adj3 (abnor$ or malform$ or anomal$)).ti,ab,kf.
417. (cloaca$ adj3 exopthalmo$).ti,ab,kf.
418. Biliary Atresia/
419. (biliary adj3 atresia$).ti,ab,kf.
420. (extrahepatic ductopen$ or extra-hepatic ductopen$ or progressive obliterative cholangiopath$).ti,ab,kf.
421. (biliary adj3 hypoplas$).ti,ab,kf.
422. (alagille$ adj3 atresia$).ti,ab,kf.
423. ((absence$ or absent$) adj3 kidney$).ti,ab,kf.
424. (potter$ adj (sequence$ or syndrome$ or disease$ or disorder$)).ti,ab,kf.
425. Oligohydramnios/
426. oligohydramn$.ti,ab,kf.
427. Multicystic Dysplastic Kidney/
428. ((kidney$ or renal) adj3 dysplas$).ti,ab,kf.
429. ((meckel$ or meckelgruber$ or gruber$) adj (syndrome$ or disease$ or disorder$)).ti,ab,kf.
430. dysencephalia splanchnocystica$.ti,ab,kf.
431. (pena-shokeir$ or penn-shokeir$).ti,ab,kf.
432. (larsen$ adj (syndrome$ or disease$ or disorder$)).ti,ab,kf.

433. Acrocephalosyndactylia/
434. acrocephalosyndactyl$.ti,ab,kf.
435. (pfeiffer$ adj (syndrome$ or disease$ or syndrome$)).ti,ab,kf.
436. Short Rib-Polydactyly Syndrome/
437. short rib$1.ti,ab,kf.
438. (saldino-noonan$ or majewski$ or verma-naumoff$ or beemer-langer$).ti,ab,kf.
439. (jeune$ adj (syndrome$ or disease$ or disorder$)).ti,ab,kf.
440. asphyxiating thoracic dysplas$.ti,ab,kf.
441. exp Chondrodysplasia Punctata/
442. chondrodysplasia punctata$.ti,ab,kf.
443. ((conradi$ or h?nermann$ or happle$) adj3 (syndrome$ or disease$ or disorder$)).ti,ab,kf.
444. Osteogenesis Imperfecta/
445. osteogenesis imperfecta.ti,ab,kf.
446. ((brittle bone or lobstein$) adj (disease$ or disorder$ or syndrome$)).ti,ab,kf.
447. Osteochondrodysplasias/
448. (spondyloepimetaphyseal or spondyloepiphyseal or spendylo metaphyseal).ti,ab,kf.
449. Hernia, Umbilical/
450. (omphalocele$ or omphalocoele$ or exomphalos).ti,ab,kf.
451. (hernia$ adj3 umbilic$).ti,ab,kf.
452. Gastroschisis/
453. gastroschis$.ti,ab,kf.
454. Ichthyosis, Lamellar/
455. (lamellar$ adj3 ichthyos$).ti,ab,kf.
456. ((harlequin$ or harloquin$) adj3 (ichthyos$ or baby or babies or f?etus$)).ti,ab,kf.
457. (ichthyosis congenita$ or ichthyosis fetalis or keratosis diffusa fetalis).ti,ab,kf.
458. exp Epidermolysis Bullosa/
459. epidermolysis bullosa$.ti,ab,kf.
460. (johanson-blizzard$ or johanna-blizzard$).ti,ab,kf.
461. Xeroderma Pigmentosum/
462. xeroderma pigmentosum.ti,ab,kf.
463. Ectodermal Dysplasia/
464. lacrimo-auriculo-dento-digital.ti,ab,kf.
465. ectodermal dysplas$.ti,ab,kf.
466. ((ladd or eec) adj (syndrome$ or disease$ or disorder$)).ti,ab,kf.
467. Sturge-Weber Syndrome/
468. (sturge-weber or encephalotrigeminal angiomatos$).ti,ab,kf.
469. Fetal Alcohol Spectrum Disorders/
470. f?etal alcohol.ti,ab,kf.
471. Pierre Robin Syndrome/
472. pierre robin$.ti,ab,kf.
473. Acrocephalosyndactylia/
474. (acrocephalosyndact$ or acrocephalopolysyndact$).ti,ab,kf.

475. ((apert$ or crouzon$ or saethre-chotzen$ or noack$ or carpenter$ or sakati-nyhan-tisdale$ or goodman$) adj (syndrome$ or disorder$ or disease$)).ti,ab,kf.
476. Fraser Syndrome/
477. (fraser$ adj (syndrome$ or disease$ or disorder$)).ti,ab,kf.
478. cryptophthalmos.ti,ab,kf.
479. (cyclopia$1 or cyclocephal$ or synophthalmi$).ti,ab,kf.
480. Goldenhar Syndrome/
481. (goldenhar$ or oculo-auriculo-vertebral).ti,ab,kf.
482. Mobius Syndrome/
483. ((m?bius$ or moebius$) adj (syndrome$ or disease$ or disorder$)).ti,ab,kf.
484. Orofaciodigital Syndromes/
485. (orofaciodigital or oro-facial-digital or oral-facial-digital or papillon-league$ or psaume$).ti,ab,kf.
486. (robin$ adj (syndrome$ or disorder$ or disease$)).ti,ab,kf.
487. (freeman-sheldon$ or distal arthrogrypos$ or craniocarpotarsal dysplas$ or craniocarpotarsal dystroph$ or canio-carpo-tarsal or windmill-vane-hand$ or whistling-face).ti,ab,kf.
488. De Lange Syndrome/
489. ((de lange$ or bushy$) adj (syndrome$ or disorder$ or disease$)).ti,ab,kf.
490. amsterdam dwarfism.ti,ab,kf.
491. (aarskog or faciodigitogenital or facio-digito-genital or facial digital genital or shawl scrotum or faciogenital or facio-genital).ti,ab,kf.
492. Cockayne Syndrome/
493. (cockayne$ or neill-dingwall$).ti,ab,kf.
494. (cerebro-oculo-facio-skeletal or cerebro-oculo-facial-skeletal).ti,ab,kf.
495. (dubowitz$ adj (syndrome$ or disease$ or disorder$)).ti,ab,kf.
496. (robinow$ or robinhow$).ti,ab,kf.
497. (f?etal face or f?etal facies or f?etal faces or acral dysostos$ or mesomelic dwarfism or covesdem$).ti,ab,kf.
498. Silver-Russell Syndrome/
499. (silver-russell$ or russell-silver$).ti,ab,kf.
500. (silver$ adj (syndrome$ or disease$ or disorder$)).ti,ab,kf.
501. ((seckel$ or harper$) adj (syndrome$ or disease$ or disorder$)).ti,ab,kf.
502. (microcephalic primordial dwarfism or bird-headed dwarf$ or virchow-seckel dwarfism).ti,ab,kf.
503. Smith-Lemli-Opitz Syndrome/
504. (smith-lemli-opitz$ or dehydrocholesterol reductase deficien$).ti,ab,kf.
505. Prader-Willi Syndrome/
506. (prader-willi$ or pradar-willi$).ti,ab,kf.
507. Rubinstein-Taybi Syndrome/
508. (rubinstein-taybi$ or rubenstein-tabyii$ or broad thumb-hallux).ti,ab,kf.
509. ((rubinstein$ or rubenstein$) adj2 (syndrome$ or disease$ or disorder$)).ti,ab,kf.
510. Nephritis, Hereditary/
511. (alport$ adj (syndrome$ or disease$ or disorder$)).ti,ab,kf.
512. (hereditary nephritis or h?emorrhagic familial nephritis).ti,ab,kf.

513. (hereditary deafness adj3 nephropath$).ti,ab,kf.
514. (h?ematuria adj3 nephropath$ adj3 deafness).ti,ab,kf.
515. Laurence-Moon Syndrome/
516. laurence-moon$.ti,ab,kf.
517. Bardet-Biedl Syndrome/
518. (bardet-biedl$ or biedl-bardet$).ti,ab,kf.
519. Zellweger Syndrome/
520. zellweger$.ti,ab,kf.
521. ((cerebrohepatorenal or cerebro-hepato-renal) adj (syndrome$ or disease$ or disorder$)).ti,ab,kf.
522. (edward$ adj (syndrome$ or disease$ or disorder$)).ti,ab,kf.
523. "trisomy 18".ti,ab,kf.
524. (patau$ adj (syndrome$ or disease$ or disorder$)).ti,ab,kf.
525. ("trisomy 13" or "trisomy D").ti,ab,kf.
526. "trisomy 22".ti,ab,kf.
527. "trisomy 9".ti,ab,kf.
528. "trisomy 10".ti,ab,kf.
529. duplication syndrome$.ti,ab,kf.
530. (("chromosome 8" or "chr 8") adj5 duplicat$).ti,ab,kf.
531. Chromosome Duplication/
532. exp X Chromosome/ab [Abnormalities]
533. exp X Chromosome/ and duplicat$.ti,ab,kf.
534. (("chromosome x" or "chr x") and duplicat$).ti,ab,kf.
535. (chromosom$ abnormality adj5 duplicat$).ti,ab,kf.
536. "tetrasomy 5p".ti,ab,kf.
537. (tetrasomy adj3 mosaic$).ti,ab,kf.
538. Chromosomes, Human, Pair 5/ and Mosaicism.mp.
539. Tetrasomy/
540. Trisomy/ and (chromosomes, human, pair 9/ or chromosomes, human, pair 10/ or chromosomes, human, pair 13/ or Chromosomes, Human, Pair 18/ or chromosomes, human, pair 22/)
541. Chromosome Deletion/ and Chromosomes, Human, Pair 4/
542. (delet$ adj5 short arm adj5 "chrom$ 4").ti,ab,kf.
543. Wolf-Hirschhorn Syndrome/
544. ((wolf-hirschhorn$ or wolff hirschorn$ or chromosome deletion dillan$ or pitt-rogers-dank$ or pitt$) adj3 (syndrome$ or disease$ or disorder$)).ti,ab,kf.
545. Cri-du-Chat Syndrome/
546. ((cri du chat$ or crying cat$ or 5p or lejeune$) adj3 (syndrome$ or disease$ or disorder$)).ti,ab,kf.
547. Jacobsen Distal 11q Deletion Syndrome/
548. ((jacobsen$ or 11q deletion) adj5 (syndrome$ or disease$ or disorder$)).ti,ab,kf.
549. exp Monosomy/ and Chromosomes, Human, Pair 9/
550. (9p minus or 9p deletion).ti,ab,kf.
551. (alfi$ adj (syndrome$ or disease$ or disorder$)).ti,ab,kf.
552. (degouchy$ or de gouchy$ or degrouchy$ or de grouchy$).ti,ab,kf.

553. distal 18q.ti,ab,kf.
554. Hypoventilation/cn [Congenital]
555. (ondine$ curse or congenital central hypoventilation or primary alveolar hypoventilation).ti,ab,kf.
556. Graft vs Host Disease/ and (Chronic Disease/ or chronic$.ti,ab,kf.)
557. (((graft vs host or graft versus host) adj (disease$ or syndrome$ or disorder)) and chronic$).ti,ab,kf.
558. exp HIV/
559. exp HIV Infections/
560. (HIV or human immunodeficiency virus$).ti,ab,kf.
561. (htlv or human t-lymphotropic virus$ or human t cell lymphotropic virus$).ti,ab,kf.
562. (acquired immune deficiency syndrome$ or acquired immunodeficiency syndrome$).ti,ab,kf.
563. (AIDS adj3 (virus$ or infection$)).ti,ab,kf.
564. (AIDS adj (related or associated)).ti,ab,kf.
565. exp Neoplasms/
566. (cancer$ or carcin$ or tumor$ or tumour$ or neoplas$ or adenocarcin$ or oncol$ or malignan$).ti,ab,kf.
567. Cystic Fibrosis/
568. (cystic fibrosis or fibrocystic or fibro-cystic or mucoviscidosis or cf).ti,ab,kf.
569. Cerebral Palsy/
570. (cerebr$ adj3 pals$).ti,ab,kf.
571. Muscle Spasticity/
572. spasticit$.ti,ab,kf.
573. Quadriplegia/
574. (spastic$ and (quadripleg$ or tetrapleg$)).ti,ab,kf.
575. exp Renal Insufficiency/
576. ((kidney$ or renal) adj3 (failure$ or insufficienc$)).ti,ab,kf.
577. (end stage adj3 (kidney or renal)).ti,ab,kf.
578. (("stage 5" or "stage V") adj3 (kidney or renal)).ti,ab,kf.
579. (ESRD or ESKD or ESRF or ESKF or CRF or CKF).ti,ab,kf.
580. or/22-579
581. Qualitative Research/
582. Interview/
583. Personal Narratives/
584. Grounded Theory/
585. Focus Groups/
586. Hermeneutics/
587. Anthropology, Cultural/
588. qualitative.af.
589. interview$.af.
590. findings.ti,ab.
591. experiences.ti,ab.
592. views.ti,ab.

593. perspective$.ti,ab.
594. beliefs.ti,ab.
595. attitude$.ti,ab.
596. narrative.ti,ab.
597. ethnograph$.ti,ab.
598. (case study or case studies).ti,ab.
599. thematic analysis.ti,ab.
600. themes.ti,ab.
601. grounded theory.ti,ab.
602. field notes.ti,ab.
603. audio record$.ti,ab.
604. focus group$.ti,ab.
605. conversation$ analys$.ti,ab.
606. descriptive stud$.ti,ab.
607. discourse analys$.ti,ab.
608. exploratory analys$.ti,ab.
609. exploratory stud$.ti,ab.
610. Hermeneutic$.ti,ab.
611. naturalistic.ti,ab.
612. phenomenolog$.ti,ab.
613. participatory.ti,ab.
614. semi structured.ti,ab.
615. key informant$.ti,ab.
616. cultural anthropology.ti,ab.
617. narration.ti,ab.
618. narrative analysis.ti,ab.
619. inductive.af.
620. content analysis.ti,ab.
621. discourse analysis.ti,ab.
622. or/581-621
623. 8 and 21 and 580 and 622
