# Supplementary material for: Experiences of fathers of children with a life-limiting condition: a systematic review and qualitative synthesis
Source: BMJ Support Palliat Care. 2021 Jun 17;13(1):15–26. doi: 10.1136/bmjspcare-2021-003019 (PMC9985706; doi:10.1136/bmjspcare-2021-003019)
Supplement: Supplementary data [file bmjspcare-2021-003019supp002.pdf]

Supplemental Material Table 1 Key Characteristics of Included Studies

Study	Aim	Year of data collection	Country	Setting	Methodology	Data collection	Data analysis	Sample	Participant demographics	Child demographics	Disease characteristics
**Cancer**											
Brody and Simmons, 2006	To explore the challenges that fathers face following their child's cancer diagnosis, how they cope with and adapt to this diagnosis.	Not stated	US	University paediatric oncology clinic, Kentucky	Qualitative descriptive design	Semi-structured interviews	Open coding	8 fathers	***Relationship***: married (5), separated/ divorced (2), single parent (1) ***Other children***: More than 1 child (7) ***Ethnicity***: Caucasian (8) ***Health***: poor/fair (4), very good/excellent (4)	***Age:*** 4-16 years (*M*= 8.2) ***Sex:*** Female (2), Male (6)	***Diagnosis:*** Acute lymphoblastic leukemia (4), T cell lymphoma (1), Ewing sarcoma (1), rhabdomyosarcoma (1), blastoma liver cancer (1), made 3 months to 2 years before study. ***Treatment:*** chemotherapy (8), radiation (4), surgical (4), receiving treatment at time of interview or completed within last 12 months.
Chamberlain, 2007Chamberlain [42]	To explore resilience in fathers of children with leukemia	Not stated	US	Leukemia & Lymphoma society	Not stated	Semi-structured interviews	Grounded theory	8 biological fathers	***Age***: 27-51 years ***Ethnicity***: Caucasian (8) ***Relationship***: married (7), divorced (1) ***Religion***: Christian (3), Catholic (2), Baptist (1), none (2)	***Age:*** 4-17 years (2-13 at diagnosis) ***Sex:*** male (5), female (3)	***Diagnosis:*** leukemia in remission ***Treatment:*** out of treatment (5), still in treatment (3)

Study	Aim	Year of data collection	Country	Setting	Methodology	Data collection	Data analysis	Sample	Participant demographics	Child demographics	Disease characteristics
									Employment: range of occupations (income $60,000-$2,000,000)		
Chesler and Parry 2001	To explore the experiences of fathers of children with cancer with a particular focus on how such experiences are influenced by gender.	1978-1998	US	Participants identified from survey data (1978-1998), focus group conducted at conference	Not stated	In-depth interviews, workshops	Deductive coding using stress framework followed by inductive coding using a grounded theory approach	167 bereaved and non-bereaved fathers	***Age:*** varies significantly* ***Education:*** varies significantly amongst sample* ***Occupation:*** varies significantly amongst sample* <br>*characteristics not described in detail	Not stated	***Treatment:*** some children still in treatment, some who had successfully completed treatment. Some children had died.
Clarke, 2005	To describe fathers' experiences of having a child with cancer, with a focus on home healthcare work concerns.	Not stated	Canada	Recruited through support groups/ partners/ researchers personal network. Child treat in geographical location in past 5 years	Not stated	Qualitative interviews	Deductive coding based on home healthcare work	16 fathers	***Age***: *M*=44; ***Relationships***: married/ long-term (15), separated (1); ***Employment:*** variety of occupations, 44% reduced work hours at diagnosis, 1 stopped working completely ***Nationality:*** English, French, Canadian, Scottish, Latin American,	***Age***: *M*=10, *M*=7 at diagnosis.	***Treatment:*** Treated within last 5 years, length of treatment *M*=26 months

Study	Aim	Year of data collection	Country	Setting	Methodology	Data collection	Data analysis	Sample	Participant demographics	Child demographics	Disease characteristics
									Portuguese, Irish Canadian		
Cluley, 2015	To explore the experiences of fathers of children with cancer and how they understand their role in their child's illness	Not stated	New Zealand	Child Cancer Foundation	Narrative inquiry- social constructionist approach	Narrative interviews	Narrative analysis	12 biological fathers	***Age:*** 33-54 years ***Ethnicity:*** New Zealand European (7), English (3), South African (1), Cook Island Māori (1)	***Age:*** 4-12 years	***Diagnosis:*** cancer including acute lymphocytic lymphoma, Burkett's Lymphoma, throat & mouth cancer, T-cell lymphoma, Wilm's tumor and Ependymoma ***Treatment:*** surgery, chemotherapy, radiotherapy-outpatients at time of study
Hill, Higgins and Dempster, 2009	To explore fathers' perceptions of their role during their child's treatment for acute lymphoblastic lymphoma	Not stated	Northern Ireland	Treating hospital	Phenomenology	Semi-structured interviews	IPA	5 fathers	***Age:*** 31-42 years **Re*lationships:*** married and living with child and partner (5) ***Other children:*** more than 1 child (4), child born during treatment (3) ***Employment:*** Full time (5)	***Age****:* 18 months-7 years at time of diagnosis;	***Diagnosis****:* ALL, in remission. ***Treatment****:* completed within last 2.5. years.
Jones and Neil-Urban, 2003	To investigate the experiences of	Not stated	US	Hospital	Group phenomenological approach	Focus groups	Grounded theory, constant	10 fathers including 1	***Age range****:* 25-56 years (mean= 40 years)	***Age:*** 3-16 years (M=1.5) ***Sex:*** male (5), female (5)	***Diagnosis:*** Cerebellum Meduloblastoma,

Study	Aim	Year of data collection	Country	Setting	Methodology	Data collection	Data analysis	Sample	Participant demographics	Child demographics	Disease characteristics
	fathers of children with cancer with a focus on the caregiving experience						comparison method, group process analysis (secondary analysis)	grandfather and 2 step-fathers	***Ethnicity***: Caucasian (9), Hispanic (1) ***Relationships:*** Married (10); ***Occupation:*** Employed (9), retired (1);		Ewings Sarcoma, Leukemia (ALL, AML), Osteogenic Sarcoma, T-cell Lymphoma ***Treatment:*** children currently in treatment. Length= 2 months-14 years (M=1.5 years)
Mojica, 2018	To explore masculinity, caregiving and coping in the context of having a child with cancer	Not stated	US	Paediatric hospital	Mixed methods	Open-ended questions	Content analysis	20 fathers	***Age range:*** 26-55 years (M=39 years) ***Relationship:*** married (11), unmarried (8), divorced (1) ***Nationality:*** Mexican (14), Ecuadorian (2), Honduran (1), Mexican-American (1), Peruvian (1), Puerto Rican (1) ***Religion:*** Roman Catholic (14), Protestant (2), Mormon (1), none (4) ***Employment:*** employed (19) (including forklift driver, assistant manager, factory worker, cook),	***Age:*** 1-18 years (*M*=2.5 years)	***Diagnosis:*** leukemia (12), osteosarcoma (2), neoplasm (6) ***Time since diagnosis:*** 3-5 months (5), 6-8 months (5), 9-12 months (4), >1 year (2), >3 years (4)

Study	Aim	Year of data collection	Country	Setting	Methodology	Data collection	Data analysis	Sample	Participant demographics	Child demographics	Disease characteristics
									unemployed (1) (9 partners unemployed) ***Education:*** 1st-8th grade (7), 9-12th grade (6), General Educational Development (1), some college (5), master's degree (1) ***Residence:*** Chicago (10), suburban Chicago (6), >40 miles from Chicago (4) ***Other children:*** only child (4), other children (2-4) (16)		
Neil-Urban and Jones 2002	To describe the experiences and coping of fathers who have a child with cancer	Not stated	US	Hospital	Group phenomenological approach	Focus groups	Thematic approach	10 fathers including 1 grandfathers and 2 step-fathers	***Age range***: 25-56 years (mean= 40 years); ***Ethnicity:*** Caucasian (9), Hispanic (1); ***Relationships:*** Married (10); ***Employment:*** Employed (9), retired (1);	***Age:*** 3-16 years (M=1.5) ***Sex:*** male (5), female (5)	***Diagnosis***: Cerebellum Meduloblastoma, Ewings Sarcoma, Leukemia (ALL, AML), Osteogenic Sarcoma, T-cell Lymphoma ***Treatment:*** children currently in treatment. Length= 2 months-14 years (M=1.5 years)

Study	Aim	Year of data collection	Country	Setting	Methodology	Data collection	Data analysis	Sample	Participant demographics	Child demographics	Disease characteristics
Nicholas et al., 2009	To examine experiences of fatherhood in the context of childhood cancer from the perspectives of fathers	Not stated	Canada	Central paediatric hospital	Grounded Theory	Semi-structured interviews	Grounded Theory	16 fathers including 14 biological fathers, 1 adoptive father and 1 step-father	***Age range:*** 20-60 years (M=43 years) ***Country of birth:*** Canada (10), Afghanistan (1), India (1), Pakistan (1), Portugal (1), Trinidad (1), US (1)	***Age:*** 1-17 years	***Diagnosis:*** leukemia or oncological disease ***Treatment:*** active treatment at time of interview
Ogg, 1997	To examine the effect of a paediatric cancer diagnosis from fathers' perspectives	Not stated	US	Haematology/ oncology outpatient clinic at medical centre	Not stated	Structured interviews	Grounded Theory	7 fathers	***Age:*** 25-43 years (*M*=36) ***Ethnicity:*** Caucasian (3), Latino (3), Asian (1) ***Education:*** high school (6), some college (5) ***Employment:*** full time (6), unemployed (1) ***Income:*** <$15,000 (1), $15,000-29,999 (1), $30,000-44,999 (1), $45,000-59,000 (3), $45,000-59,000 (2)	Not stated	***Diagnosis:*** Acute Lymphoblastic Lymphoma (ALL) (6), spinal chord tumour (1) ***Time since diagnosis:*** 2- 8 weeks
Robinson et al., 2019a	To explore the paternal roles, responsibilities, strengths, challenges, personal growth and	December 2018- February 2019	US	Paediatric Hospital	Not stated	Semi-structured interviews	Semantic content analysis	4 fathers	***Ethnicity:*** Caucasian (4) ***Education:*** completed college (2), some college (2) ***Occupation:*** manual work (2), office work (2)	***Age:*** 5-14 years (*M*= 9.25 years) ***Sex:*** male (2), female (2)	***Diagnosis:*** brain tumour, 3-168 months since diagnosis (*M*=47.8 months) ***Treatment:*** in hospital at time of

Study	Aim	Year of data collection	Country	Setting	Methodology	Data collection	Data analysis	Sample	Participant demographics	Child demographics	Disease characteristics
	support needs of fathers of children with brain tumors.								***Primary caregiver:*** mother (1), father (1), both (2)		interview (3), outpatient (1)
Wolff et al., 2010	To look at how fathers describe their experiences and challenges, sources of support and coping and unique challenges described by single fathers and those from racial and ethnic minority groups.	Not stated	US	Paediatric Hospital	Life-story method	Semi-structured interviews	Narrative analysis with multiple case studies	15 fathers- primary medical caretakers	***Age range:*** 32-56 years (M=44)	Not stated	***Diagnosis***: Cancer or SCD diagnosed at least 6 months prior to interview. Sickle cell disease, leukemia, brain tumour, other tumour.
Wolff et al., 2011	To understand why fathers assume the primary caregiving role to their child with a life-threatening illness; to examine how	Not stated	US	Paediatric Hospital	Life-story method	Semi-structured interviews	Narrative analysis with multiple case studies	As above	As above	As above	As above

Study	Aim	Year of data collection	Country	Setting	Methodology	Data collection	Data analysis	Sample	Participant demographics	Child demographics	Disease characteristics
	they describe their reasons for assuming this role and how race, ethnicity and socioeconomic status shape this decision										
Wills, 2009	To describe the experiences coping strategies of fathers of children with acute lymphocytic leukemia	Not stated	China	Paediatric oncology ward, large teaching hospital, Hong Kong	Not stated	Semi-structured interviews	Qualitative data analysis using matrix system	8 fathers	***Age:*** *M*=38 years ***Number of other children:*** 1(1), 2(4), 4(3) ***Education:*** Elementary school (2), High school (3), some university (1), university (2) ***Employment:*** Employed (8) (Hairdresser, driver, businessman, police officer, insurance)	***Age range:*** 9 months-14 years ***Sex:*** male (4), female (4)	***Diagnosis:*** acute lymphoblastic lymphoma
**Congenital Heart Defect**											
Bright et al., 2013	To examine the relationship between fathers and their infant with CHD	Not stated	Australia	Paediatric hospital	Mixed methods	Interviews	Qualitative analysis	63 fathers	***Age range:*** 9.63-48.46 years (*M*=34.33 years) ***Education:*** 64% fathers completed secondary school, 30% completed university education. ***Occupation:*** 5%	***Age range:*** 42-202 days (*M*=81.31 days) ***Sex:*** male (34), female (29) ***Time between discharge & interview:*** *M*=61.84 days ***Length of stay:*** 1-14 days (27%), 29-42 days (16%), ≥ 43 days (25%)	***Diagnosis:*** congenital heart defect

Study	Aim	Year of data collection	Country	Setting	Methodology	Data collection	Data analysis	Sample	Participant demographics	Child demographics	Disease characteristics
									fathers reported that government benefit was their main source of income. ***SES:*** The Daniel Scale of Occupational Prestige (1= high SES, 7= low SES). Average 4.35		
Bruce, Lindh and Sundin, 2016	To explore the experiences and meaning of support from the perspectives of fathers of children with congenital heart defects	2009	Sweden	Paediatric cardiac outpatient clinic	Phenomenological-hermeneutic	Narrative interviews	Phenomenological-hermeneutic method for interpreting narrative interviews	5 fathers	***Age range:*** 24-47 years (M=37.8 years) ***Relationship:*** married (3), cohabiting (2) ***Employment:*** Employed (5) ***Other children:*** More than one child (2)	***Age:*** 3-12 years (*M*=6.6 years), born between 1996 & 2006 ***Sex:*** male (4), female (1)	***Diagnosis:*** Aortic stenosis, hypoplastic left heart syndrome, tetralogy of Fallot, pulmonary atresia
Clark and Miles, 1999	To explore the experiences of fathers whose infants were diagnosed with severe congenital heart disease	Not stated	US	NICU, ICU, paediatric ICU	Not stated	Longitudinal semi-structured interviews at diagnosis and 12 months post-diagnosis	Content analysis	8 fathers	***Age range***: 23-40 years (M=28 years) ***Relationship:*** married (7) ***Ethnicity:*** white (6), Asian (1), African American (1) ***Education:*** high school (8), college/some college (5), graduate school (2)	***Age:*** infant-18 months	***Diagnosis:*** tetralogy of Fallot, atrial ventricular septal defect, coarctation of the aorta, supraventricular tachycardia, transportation of the great vessels, double-outlet right ventricle, pulmonary atresia ***Treatment:***

Study	Aim	Year of data collection	Country	Setting	Methodology	Data collection	Data analysis	Sample	Participant demographics	Child demographics	Disease characteristics
									Other children: 1 child (6), 2 children (2)		hospitalised, technology dependent at time of enrolment, surgery (7) including heart transplant (1)
Gower et al., 2017	To explore the lived experiences of fathers of children with a congenital heart defect	Not stated	UK	Regional clinic	Not stated	Semi-structured interviews	IPA	6 fathers	***Age:*** 28-49 years ***Relationship:*** lived with child & partner (6) ***Other children:*** first time father (2), other children (4) ***Employment:*** full-time (6)	***Age:*** 7 months-2 years	***Diagnosis:*** congenital heart hefect ***Treatment:*** 1-3 corrective procedures- several awaiting further surgeries
Robinson, 2019b	To understand the needs, role understanding and resiliency in fathers of children receiving cardiac care with a new palliative care referral	Not stated	US	Children's hospital	Not stated	Interviews	Semantic content analysis	10 fathers	***Ethnicity:*** African American (1), Caucasian (8), Hispanic (1) ***Lives with child:*** yes (7), no (3) ***Number of other children in home:*** 0 (3), 1 (3), 2 (2), 3 (2) ***Marital status:*** married (7), engaged (2), single (1) ***Education:*** high school diploma (4), some college (3), college completion (3)	***Sex:*** male (8), female (2) ***Age:*** 7 months to 7 years (*M*=19 months).	***Diagnosis:*** heart condition requiring surgery, diagnosed an average of 17 months prior to interview. Interviews within two weeks of surgery. 8 in ICU at time of interview.

Study	Aim	Year of data collection	Country	Setting	Methodology	Data collection	Data analysis	Sample	Participant demographics	Child demographics	Disease characteristics
									Employment: labourer (5), sales/retail (2), stay at home father (1), teacher (2) ***Primary caregiver:*** mother (4), father (1), both (5)		
**Cystic fibrosis**											
Hayes and Savage, 2008	To examine the emotional impact of caring for a child with CF from fathers' perspectives	Not stated	Ireland	Cystic fibrosis centre	Not stated	Qualitative interviews	Thematic content analysis	8 biological fathers	***Education:*** at least second level education (8) ***Relationship:*** married (8) ***Employment:*** employed (8)	***Age:*** 18 months-6 years	***Diagnosis:*** cystic fibrosis, diagnosed for at least 1 year
Priddis et al., 2010	To explore fathers' perceptions of the familial impact of their infant's CF diagnosis	Not stated	Australia	Paediatric care provider	Not stated	Semi-structured interviews	Content analysis	15 fathers	***Age:*** 28-52 years (M=37.33 years) ***Relationship:*** 'intact' family (15) ***Residence:*** metropolitan area (10), country (5) ***Other children:*** other children at home (11)	***Age:*** 0.75-5.75 years (*M*=3.4 years)	***Diagnosis:*** cystic fibrosis, diagnosed at least 6 months prior to study, mean age at diagnosis 5.6 weeks
Shardonofsky et al., 2019	To explore fathers' perspectives of caring for a child with cystic fibrosis	Not stated	US	Cystic fibrosis centres	Descriptive Phenomenology	Semi-structured interviews	Descriptive phenomenology	20 fathers	***Age:*** 26-54 years (*M*=38.7 years) ***Education:*** High school (2), some college (9), Bachelor's degree (7), Master's degree (1), PhD (1)	***Age:*** 5-17 years (*M*=7.6 years) ***Sex:*** male (14), female (6)	***Genotype:*** DF508/DF508 (14), other (6)

Study	Aim	Year of data collection	Country	Setting	Methodology	Data collection	Data analysis	Sample	Participant demographics	Child demographics	Disease characteristics
									Employment: Full time employed (19), unemployed (1) ***Health insurance:*** Private through employer (16), private bought outright (1), Medicaid/ Medicare (3) ***Ethnicity:*** White (16), Hispanic (3), Asian/ Indian (1) ***Married to mother of child with CF:*** Yes (18), No (2) (both co-parenting) ***Number of children with CF:*** 1 (16), 2 (3), 3 (1)		
**Genetic conditions**											
Rivard and Mastel-Smith, 2014	To describe the experiences of fathers who have children diagnosed with a genetic disorder.	Recruitment April-October 2012	US	Identified via nurses, physicians, genetic counsellors, snowball sampling, email, internet, word-of-mouth	Phenomenology	Semi-structured interviews	IPA	6 biological fathers	***Education:*** college (6) ***Ethnic group:*** White (6) ***Relationship:*** married (6) ***Income:*** $20,000-115,000 per year	Not stated	***Diagnosis:*** Genetic Disorder (Galactosemia, Prader-Willi-Syndrome, Isovaleric acidemia, very-long-chain-acyl-CoA de-hydrogenase deficiency (VCLAD), Downs Syndrome, osteogenesis

Study	Aim	Year of data collection	Country	Setting	Methodology	Data collection	Data analysis	Sample	Participant demographics	Child demographics	Disease characteristics
											imperfecta, type 1)<br><br>Time since diagnosis ranged from 15 months to 13 years.
**Life-limiting conditions**											
Bailey-Pearce et al., 2018	To explore the experiences of fathers of children with a LLC and to examine how their attachment strategies influence these experiences.	Not stated	UK	Identified via community paediatric nursing team	Narrative approach	Narrative interviews	Narrative analysis	7 biological fathers	***Age:*** 27-54 years<br>***Relationships:*** In relationship with child's mother (5), separated since diagnosis (2)<br>***Other:*** 1 father had 2 children, both with a LLC	Not stated	***Diagnosis:*** diagnosed with a LLC for at least 1 year
Davies et al., 2013	To explore fathers' perspectives of the care received from healthcare professionals during their child's illness and death	Not stated	US	Paediatric palliative care settings	Grounded Theory	Semi-structured interviews, field notes, genograms, reflexive journaling, interview debriefing	Open coding, constant comparative analysis	60 bereaved fathers including 55 biological fathers, 3 step-fathers, 1 uncle & 1 grandfather/	***Age:*** 24-60 years<br>***Relationship:*** married (53), divorced (5), single (1), widowed (1)<br>***Education:*** high school education (55)<br>***Occupation:*** High average income	***Age at time of death:*** 21 years or younger	***Diagnosis:*** Life-threatening illness

Study	Aim	Year of data collection	Country	Setting	Methodology	Data collection	Data analysis	Sample	Participant demographics	Child demographics	Disease characteristics
								adoptive father			
Davies et al., 2010	To provide a description and theoretical explanation of fathers' experiences of their child's life-limiting illness	Not stated	US	Paediatric hospice home care program	Grounded Theory	In-depth unstructured interviews	Grounded Theory	8 bereaved fathers	***Age:*** 34-39 years ***Relationship:*** married (8) ***Ethnicity:*** Caucasian (7), African American (1) ***Religion:*** Protestant (3), Catholic (3), Jewish (1) ***Employment:*** employed (6), student (1), absence of leave (1), varied incomes.	***Age at time of death:*** 3 months- 14 years ***Sex:*** male (4), female (4)	***Diagnosis:*** spinal muscular atrophy (2), Tay Sachs (1), cancer (5)
Nicholas et al., 2016	To explore father's experience and support provision preferences in relation to their child's life-limiting illness	Not stated	Canada	Tertiary level paediatric hospitals	Grounded Theory	Semi-structured interviews	Grounded Theory	18 fathers including 6 bereaved and 12 non-bereaved	***Relationship:*** married (17), divorced (1) ***Employment:*** employed (17), unemployed (1) ***Education***: high school (1), college or university (12), post-graduate or professional degree (2) ***Employment***: Full time (16), unemployed (1), income range $40,000->$120,000	***Age:*** <18 years ***Birth year:*** 1993-2007	***Diagnosis (primary):*** Cerebral palsy, hypoplastic left heart syndrome, epilepsy (Dravet syndrome), metacromatic leukodystrophy, pulmonary hypertension, Cystic fibrosis, Cancer (Ewing sarcoma, Neuroblastoma, Rhabdomyosarcoma, lymphoma, Neuroectodermal

Study	Aim	Year of data collection	Country	Setting	Methodology	Data collection	Data analysis	Sample	Participant demographics	Child demographics	Disease characteristics
									Religion: Catholic (6), Christian (6), Christian, Islamic (1), Islamic (1), Jehovah's Witness (1), Spiritual (1)		tumour, osteogenic sarcoma, t-cell leukemia)- diagnosed at least 6 months prior to study
Rigby, 2013	To explore the experiences and grief of fathers following the loss of their child to a life-limiting condition.	Not stated	US	Recruitment across 3 states	Secondary analysis	Semi-structured, in-depth and follow-up interviews	Qualitative analysis	24 bereaved fathers	***Age:*** *M*=43 ***Ethnicity:*** Caucasian (15), Mexican/ Latino (4), Asian (2), African- American (2), Indian (1) ***Religion:*** Catholic (9), Protestant (9), No religion (5), Hindu (1)	***Age at time of death:*** *M*=7.6 ***Sex:*** Female (12), Male (12) ***Years since death:*** 3 months- 3.1 years (*M*=1.7 years)	***Diagnosis:*** chronic, genetic or traumatic life-limiting condition
Ware and Raval, 2007	To investigate the experiences of fathers of children with a life-limiting illness and how it has affected them and their family relationships.	Not stated	UK	Recruited via several sources (newsletter, parent support groups, voluntary organisations)	Phenomenology	Semi-structured interviews	Interpretive phenomenological analysis	8 fathers	***Ethnicity:*** Caucasian (8) ***Relationship:*** lived with child's mother (5), primary carer for child (0) ***Religious beliefs:*** Christian	Not stated	***Diagnosis:*** category 3 conditions (progressive, no cure, palliative treatment may extend over many years)
**Neurological conditions**											
Applebaum and	To examine the experiences of	Not stated	US	Referred via healthcare	Phenomenology	Interviews	Thematic analysis	6 biological fathers	***Age:*** 25-60 years (median 42.5);	***Age:*** 5-27 years	***Diagnosis:*** Severe cerebral palsy

Study	Aim	Year of data collection	Country	Setting	Methodology	Data collection	Data analysis	Sample	Participant demographics	Child demographics	Disease characteristics
Smolowitz, 2012	fathers of children with severe cerebral palsy.			staff at residential facility					***Ethnicity***: Caucasian (5), Hispanic (1) ***Relationship:*** married (5), remarried to new partner (1)	***Residence:*** school facility (4), at home (2) ***Sex:*** male (3), female (3)	
Lucca and Petean, 2016	To understand the experiences of fathers of children with Duchenne Muscular Dystrophy (DMD)	Not stated	Brazil	Association of Muscular Dystrophy in Ribeirão Preto	Not stated	Semi-structured interviews	Content analysis	8 fathers	***Age:*** 35-65 years ***Relationship:*** married (10) ***Employment:*** salaries 3-10 times the minimum wage; ***Other children:*** 2 children (7), 4 children (1), 2 children with DMD (1)	***Age:*** 10 years or older; ***Sex:*** male (10)	***Diagnosis:*** Duchenne Muscular Dystrophy (DMD)
