# Supplementary material for: Experiences of fathers of children with a life-limiting condition: a systematic review and qualitative synthesis
Source: BMJ Support Palliat Care. 2021 Jun 17;13(1):15–26. doi: 10.1136/bmjspcare-2021-003019 (PMC9985706; doi:10.1136/bmjspcare-2021-003019)
Supplement: Supplementary data [file bmjspcare-2021-003019supp003.pdf]

Supplemental Material Quality Appraisal Table for Included Studies

Table a; quality appraisal of included studies using modified CASP tool

	Cancer															CHD
Question	Study	Brody, 2007	Chamberlain, 2007	Chesler, 2001	Clarke, 2005	Cluley, 2015	Hill, 2009	Jones, 2003	Mojica, 2016	Neil-Urban, 2002	Nicholas, 2009	Ogg, 1997	Robinson, 2019	Wolff, 2010	Wolff, 2011	Wills, 2009	Bright, 2016
	Rating	Medium	Medium	Medium	Low	High	High	Medium	Low	Medium	High	Medium	Medium	High	High	Medium	Low
1. Was there a clear statement of the aims of the research?		Yes	Yes	Yes	Yes	Yes	Yes	Yes	Yes	Yes	Yes	Yes	Yes	Yes	Yes	Yes	Yes
2. Is the qualitative methodology appropriate?		Yes	Yes	Yes	Yes	Yes	Yes	Yes	Yes	Yes	Yes	Yes	Yes	Yes	Yes	Yes	Yes
3. Was the research design appropriate to address the aims of the research?		Yes	Yes	Somewhat	Yes	Yes	Yes	Yes	Yes	Yes	Yes	Yes	Yes	Yes	Yes	Yes	Yes
4. Are the studies theoretical underpinnings clear, consistent and conceptually coherent?		No	No	No	No	Yes	Yes	No	Can't tell	No	Yes	Yes	No	Some-what	Some-what	No	No
5. Was the recruitment strategy appropriate to the aims of the research?		Yes	Yes	Yes	Yes	Yes	Yes	Yes	Yes	Yes	Yes	Yes	Yes	Yes	Yes	Yes	Yes
6. Was the data collected in a way that addressed the research issue?		Yes	Yes	Somewhat	Yes	Yes	Yes	Yes	Yes	Yes	Yes	Yes	Somewhat	Yes	Yes	Some-what	Some-what
7. Has the relationship between researcher and participants been adequately considered?		Can't tell	Can't tell	Somewhat	Can't tell	Yes	Yes	Can't tell	Can't tell	Can't tell	Yes	Can't tell	Somewhat	Yes	Yes	Can't tell	No
8. Have ethical issues been taken into consideration?		Yes	Yes	Yes	Can't tell	Yes	Yes	Can't tell	Yes	Can't tell	Yes	Yes	Somewhat	Can't tell	Can't tell	Some-what	Can't tell
9. Was the data analysis sufficiently rigorous?		Yes	Yes	Yes	Somewhat	Yes	Yes	Yes	Somewhat	Yes	Yes	Yes	Somewhat	Yes	Yes	Some-what	Can't tell
10. Is there a clear statement of findings?		Yes	Yes	Yes	Yes	Yes	Yes	Yes	Yes	Yes	Yes	Yes	Yes	Yes	Yes	Yes	Yes

Question		Cancer															CHD
	Study	Brody, 2007	Chamberlain, 2007	Chesler, 2001	Clarke, 2005	Cluley, 2015	Hill, 2009	Jones, 2003	Mojica, 2016	Neil-Urban, 2002	Nicholas, 2009	Ogg, 1997	Robinson, 2019	Wolff, 2010	Wolff, 2011	Wills, 2009	Bright, 2016
	Rating	Medium	Medium	Medium	Low	High	High	Medium	Low	Medium	High	Medium	Medium	High	High	Medium	Low
11. How valuable is the research?		a, b, c*	a, b, c*	a, b, c*	a, c*	a, b, c*	a, b, c*	a, b, c*	a, b, c*	b*	a, b, c*	a, b, c*	a, b , c*	a, b, c*	a, b, c*	a, b, c*	a, b, c*

*a. Findings considered in relation to existing research
b. Discussion relating to implications of findings upon practice or policy
c. Identification of areas in which further research is necessary

Table b; quality appraisal of included studies using modified CASP tool continued

Congenital Heart Defect						Cystic Fibrosis			Genetic conditions	Life-limiting conditions						Neurological conditions	
Question	Study	Bruce, 2016	Clark, 1999	Gower, 2016	Robinson, 2019	Hayes, 2008	Priddis, 2010	Shardo-nofsky, 2009	Rivard, 2014	Bailey-Pearce, 2017	Davies, 2013	Davies, 2004	Nicholas, 2016	Rigby, 2012	Ware, 2007	Applebaum, 2012	Lucca, 2016
	Rating	High	Medium	High	Low/Medium	High	Low	Medium	High	High	High	High	High	Medium	High	High	Low/Medium
1. Was there a clear statement of the aims of the research?		Yes	Yes	Yes	Yes	Yes	Yes	Yes	Yes	Yes	Yes	Yes	Yes	Yes	Yes	Yes	Yes
2. Is the qualitative methodology appropriate?		Yes	Yes	Yes	Yes	Yes	Yes	Yes	Yes	Yes	Yes	Yes	Yes	Yes	Yes	Yes	Yes
3. Was the research design appropriate to address the aims of the research?		Yes	Yes	Yes	Yes	Yes	Yes	Yes	Yes	Yes	Yes	Yes	Yes	Yes	Yes	Yes	Yes
4. Are the studies theoretical underpinnings clear, consistent and conceptually coherent?		Yes	No	Some-what	Can't tell	No	No	Yes	Yes	Yes	Some-what	Yes	Somewhat	Some-what	Yes	Yes	No
5. Was the recruitment strategy appropriate to the aims of the research?		Yes	Yes	Yes	Yes	Yes	Yes	Yes	Yes	Yes	Yes	Yes	Yes	Yes	Yes	Yes	Yes
6. Was the data collected in a way that addressed the research issue?		Yes	Yes	Yes	Somewhat	Yes	Yes	Yes	Yes	Yes	Yes	Yes	Yes	Yes	Yes	Yes	Yes
7. Has the relationship between researcher and participants been adequately considered?		Yes	Can't tell	Some-what	Can't tell	Can't tell	Some-what	Yes	Can't tell	Yes	Can't tell	Some-what	Somewhat	Some-what	Yes	Yes	Can't tell
8. Have ethical issues been taken into consideration?		Yes	Can't tell	Yes	Can't tell	Yes	Some-what	Some-what	Some- what	Yes	Some-what	Yes	Yes	Some-what	Yes	Can't tell	Can't tell
9. Was the data analysis sufficiently rigorous?		Yes	Somewhat	Yes	Yes	Yes	Yes	Some-what	Yes	Yes	Yes	Yes	Yes	Some-what	Yes	Yes	Some-what
10. Is there a clear statement of findings?		Yes	Yes	Yes	Yes	Yes	Yes	Yes	Yes	Yes	Yes	Yes	Yes	Yes	Yes	Yes	Yes
11. How valuable is the research?		a,b*	a, b, c*	a, b, c*	a, b*	a, b, c*	a, b, c*	a, b*	a, b, c*	a, b, c*	a, b c*	a, b, c*	a, b, c*	a, b, c*	a, b, c*	a, b, c	a, b, c*

*a. Findings considered in relation to existing research
b. Discussion relating to implications of findings upon practice or policy
c. Identification of areas in which further research is necessary
